# Evolution of a Novel Antiviral Immune-Signaling Interaction by Partial-Gene Duplication

**DOI:** 10.1371/journal.pone.0137276

**Published:** 2015-09-10

**Authors:** Bryan Korithoski, Oralia Kolaczkowski, Krishanu Mukherjee, Reema Kola, Chandra Earl, Bryan Kolaczkowski

**Affiliations:** 1 Department of Microbiology and Cell Science, Institute of Food and Agricultural Sciences, University of Florida, Gainesville, FL, United States of America; 2 Whitney Laboratory for Marine Bioscience, University of Florida, St. Augustine, FL, United States of America; 3 Department of Food Science and Human Nutrition, University of Florida, Gainesville, FL, United States of America; 4 Department of Biology, University of Florida, Gainesville, FL, United States of America; INRA, FRANCE

## Abstract

The RIG-like receptors (RLRs) are related proteins that identify viral RNA in the cytoplasm and activate cellular immune responses, primarily through direct protein-protein interactions with the signal transducer, IPS1. Although it has been well established that the RLRs, RIG-I and MDA5, activate IPS1 through binding between the twin caspase activation and recruitment domains (CARDs) on the RLR and a homologous CARD on IPS1, it is less clear which specific RLR CARD(s) are required for this interaction, and almost nothing is known about how the RLR-IPS1 interaction evolved. In contrast to what has been observed in the presence of immune-modulating K63-linked polyubiquitin, here we show that—in the absence of ubiquitin—it is the first CARD domain of human RIG-I and MDA5 (CARD1) that binds directly to IPS1 CARD, and not the second (CARD2). Although the RLRs originated in the earliest animals, both the IPS1 gene and the twin-CARD domain architecture of RIG-I and MDA5 arose much later in the deuterostome lineage, probably through a series of tandem partial-gene duplication events facilitated by tight clustering of RLRs and IPS1 in the ancestral deuterostome genome. Functional differentiation of RIG-I CARD1 and CARD2 appears to have occurred early during this proliferation of RLR and related CARDs, potentially driven by adaptive coevolution between RIG-I CARD domains and IPS1 CARD. However, functional differentiation of MDA5 CARD1 and CARD2 occurred later. These results fit a general model in which duplications of protein-protein interaction domains into novel gene contexts could facilitate the expansion of signaling networks and suggest a potentially important role for functionally-linked gene clusters in generating novel immune-signaling pathways.

## Introduction

The evolution of novel molecular interactions can have profound implications for the organism, including changing how it develops, responds to environmental challenges and interacts with symbionts and pathogens. Understanding how new molecular interactions arise, establish themselves as distinct from existing interactions, and change over evolutionary time therefore provides a reductionist foundation from which to begin answering fundamental questions regarding the evolution of organsimal complexity and novelty [1].

The RIG-like receptors (RLRs) RIG-I and MDA5 are cytoplasmic proteins that identify viral RNA and initiate cellular immune responses by interacting with the signal-transducing protein, IPS1 [2–4]. The RLR-IPS1 interaction initiates a series of signaling events that results in production of type I interferons and proinflammatory cytokines. RLR-IPS1 immune signaling is mediated by a direct physical interaction between twin N-terminal caspase activation and recruitment domains (CARDs) on the RLR and the N-terminal CARD of IPS1 [5, 6]. The third RLR, LGP2, lacks CARD signaling domains and appears to regulate antiviral immune responses through various mechanisms [7–9].

All three RLRs and IPS1 have been confidently identified in a wide variety of vertebrate genomes, suggesting that the RLR-IPS1 signaling pathway was present in the ancestral vertebrate. We have recently shown that full-length RLRs were present in the earliest metazoa [10], and IPS1 has been tentatively identified in the echinoderm, *Strongylocentrotus purpuratus*, suggesting that the RLR-IPS1 interaction may pre-date the vertebrate lineage [11]. Although the CARD domains of RLRs and IPS1 appear homologous [12], very little is known about how the RLR-IPS1 interaction evolved.

Here we use a combination of genomics, phylogenetics, structural modeling and molecular-functional characterization of ancestral and extant RLR and IPS1 CARD domains to determine when and how direct RLR-IPS1 interactions arose and initially diversified. In contrast to what has been observed in the presence of immune-modulating polyubiquitin chains [13], we find that the first CARD domains of human RIG-I and MDA5 bind directly to IPS1 CARD in the absence of ubiquitin. Evolutionary analysis suggests that RLR and IPS1 CARDs diversified in early deuterostomes, probably through a series of tandem, partial-gene duplication events. Functionally distinct RLR-IPS1 signaling pathways emerged coincident with early deuterostome CARD proliferation, potentially driven by adaptive coevolution between RLR and IPS1 CARDs. Although functionally distinct RIG-I CARD1 and CARD2 domains evolved early, functional specificity of MDA5 CARD domains appears to have arisen at a later time.

## Results and Discussion

### Human RLR CARD1s Interact Directly with IPS1 CARD when Ubiquitin Is Absent

**Fig 1:**
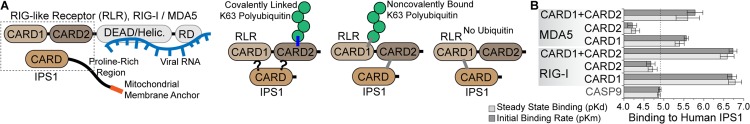
In the absence of ubiquitin, the RIG-like receptors (RLRs), RIG-I and MDA5, bind their signaling partner, IPS1, via a direct interaction between the first RLR CARD domain (CARD1) and the CARD domain of IPS1. (A) After binding viral RNA, RIG-I and MDA5 interact directly with IPS1 via CARD-CARD interactions. K63 Polyubiquitin chains—either covalently linked or noncovalently bound to RLR CARDs—can potentiate RLR-IPS1 signaling. In the case of noncovalent polyubiquitin binding, studies suggest RIG-I CARD2 interacts with IPS1 (bottom middle). Our results suggest that, in the absence of ubiquitin, RLR CARD1 interacts with IPS1 (bottom right). (B) We measured the kinetics of human RIG-I and MDA5 CARD1, CARD2 and CARD1+2 domains bound to IPS1 CARD in vitro (see Materials and Methods) (S1 Fig). We plot the mean and standard error in–log-transformed steady-state dissociation (pKd) and initial binding rate (pKm), with longer bars indicating tighter binding (S2 Fig). Human CASP9 CARD was used as a negative control to indicate an approximate level of nonspecific binding (dotted line); CASP9 is the CARD domain most closely related to RLR/IPS1 CARDs (Fig 3), and direct interactions between RLRs and CASP9 have not been reported.

Analyses of cellular immune-signaling in mammals have demonstrated convincingly that the twin N-terminal CARD domains of the RIG-like receptors (RLRs), RIG-I and MDA5, interact directly with the N-terminal CARD domain of IPS1 to initiate antiviral immune responses [5, 6]. Further studies have suggested that K63-linked polyubiquitin chains—either covalently linked to CARD2 or bound noncovalently—may potentiate RLR-based immune signaling [14, 15]. Recent structural and mutational studies suggest that—in the presence of noncovalently bound polyubiquitin—RIG-I CARD2 interacts directly with IPS1’s CARD domain, whereas RIG-I CARD1 helps to form a homotetrameric RLR complex [13, 16] (Fig 1A).

We expressed and purified human RIG-I CARD1 (amino acid positions 1–87), CARD2 (88–172), and CARD1+2 domains as well as MDA5 CARD1 (1–96) CARD2 (97–204) and CARD1+2 domains and measured their binding kinetics to human IPS1 CARD (1–92) in vitro (S1 Fig). In the absence of polyubiquitin, we found that RIG-I and MDA5 CARD1 and CARD1+2 domains bound to IPS1 CARD with high affinity, whereas CARD2 domains did not (Fig 1B and S2 Fig). Human RIG-I CARD1 bound IPS1 with the strongest affinity (dissociation constant, Kd = 3.31e^-8^ M; initial binding rate, Km = 3.89e^-8^ M), ~70-times stronger than human caspase9 CARD, which we used as a negative control (*p*<0.006). Human MDA5 CARD1 also bound IPS1 better than the negative control (MDA5 CARD1 Kd = 8.13e^-7^ M, Km = 5.50e^-7^ M, *p*<0.013), but not as strongly as RIG-I CARD1 (*p*<0.004). Interestingly, we found that neither RIG-I nor MDA5 CARD2 domains bound IPS1 CARD more tightly than the negative control (*p*>0.16). However, the presence of CARD2 domains did not interfere with RLR-IPS1 binding by CARD1, as both RIG-I and MDA5 CARD1+2 domains bound IPS1 with the same affinity as their respective CARD1 domains (*p*>0.37).

Our analyses used flag-tagged IPS1 proteins to facilitate binding measurements (see Materials and Methods). Although unlikely, the presence of the flag polypeptide could alter IPS1’s binding kinetics in vitro. However, similar results were obtained using either a maltose-binding-protein IPS1 tag (S3 Fig) or flag-tagged RLR proteins bound to untagged IPS1 tag (S3 Fig), arguing against this interpretation.

We divided RLR CARD1+CARD2 sequences into CARD1 and CARD2 using functional domains annotated in the Pfam, CDD and SMART databases (see Materials and Methods). Dividing CARD1+2 into individual functional domains that do not precisely map to structural domain boundaries could introduce binding artifacts by altering the folding of individual functional domains, compared to the CARD1+2 multidomain construct. To examine this possibility, we measured IPS1 CARD binding of structurally-determined RIG-I CARD1 (amino acid positions 1–93) and CARD2 (102–188) as well as MDA5 CARD1 (1–101) and CARD2 (113–204) constructs (S1 Fig). We found no difference in the pattern of which RLR CARDs bound IPS1, whether CARD boundaries were identified using sequence or structural data. Structurally-determined RIG-I and MDA5 CARD1 (excluding the CARD1-CARD2 linker) bound IPS1 better than the negative control (*p*<0.021 for RIG-I; *p*<0.034 for MDA5), while CARD2 domains (excluding the interdomain linker) did not (*p*>0.41) (S5 Fig), suggesting that artifacts related to domain boundary identification or inclusion/exclusion of the CARD1-CARD2 linker are unlikely to have a major impact on our results.

### Protein-Protein Docking Predicts the RIG-I CARD1 –IPS1 Interface

In the presence of ubiquitin, structural and mutational studies suggest that RIG-I CARD2 interacts with IPS1 CARD through a variety of interfaces to form a large-scale helical complex [13]. Although no structure of an RLR CARD bound to IPS1 in the absence of ubiquitin has been determined, early studies of IPS1 CARD crystalized in isolation suggested that IPS1 might bind its signaling partners through an α1-α3-α4 interface [12]. In fact, the α1-α3-α4 surface of IPS1 was observed to interact with the α2-α3 surface of RIG-I CARD2 in the helical complex [13].

**Fig 2:**
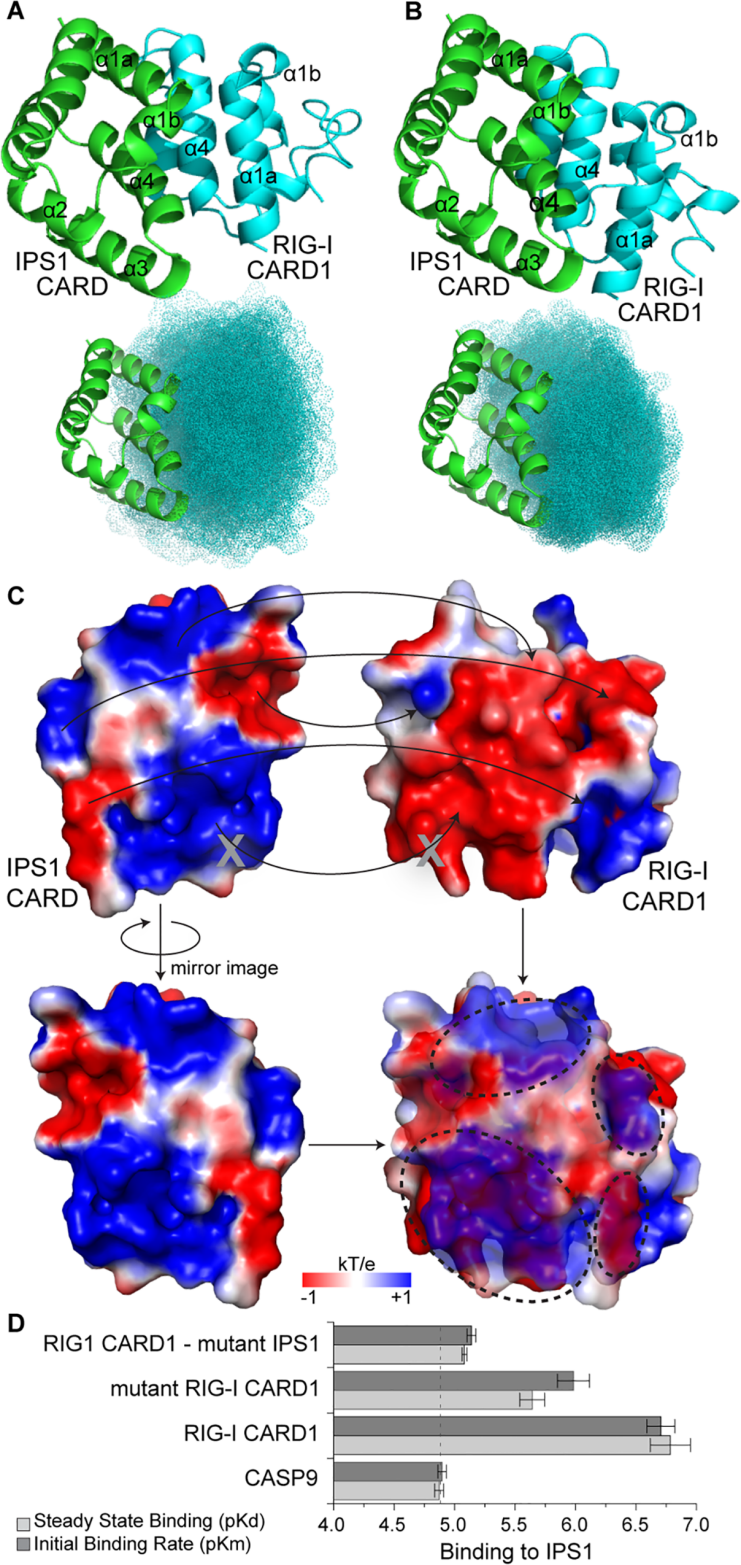
Structure-based protein-protein docking suggests RIG-I CARD1 interacts with IPS1 CARD through a novel antiparallel α1-α3-α4 interface. We used ClusPro (A) and Dot2 (B) to predict the most likely structural orientation of human RIG-I CARD1 bound to IPS1 (see Materials and Methods). We show the best-scoring complex (top panels) as well as the 20 top-scoring orientations (bottom panels) from each analysis. (C) We calculated the surface electrostatic charge (kT/e) of human RIG-I CARD1 and IPS1 CARD (see Materials and Methods). Top panel displays IPS1 (left) and RIG-I CARD1 (right) in the same antiparallel α1-α3-α4 orientation as A and B. Arrows indicate regions of inferred shape and electrostatic complementarity. Gray Xs indicate locations of mutations examined in D. In the bottom panel, IPS1 is shown in mirror image and made 35% transparent; regions of electrostatic complementarity appear purple in the overlay (also highlighted by dotted ovals). (D) We created mutant RIG-I CARD1 (Glu66,67—Arg) and IPS1 (Arg66,67—Glu) and measured the affinities with which mutant proteins bind their wild-type partners. We plot the mean and standard error–log steady-state dissociation (pKd) and initial binding rate (pKm) of each interaction (S6 Fig). Wild-type RIG-I CARD1 and CASP9 interacting with wild-type IPS1 were used as positive and negative controls, respectively (Fig 1B).

We predicted the structural interfaces between human RLR CARD1s and IPS1 CARD using protein-protein docking (see Materials and Methods). We found that a novel antiparallel α1-α3-α4 binding interface between RIG-I CARD1 and IPS1 CARD was consistently recovered in our docking analyses and was supported by additional manual examination and mutation experiments (Fig 2). Four of the top 5 (7 of the top 10) RIG-I—IPS1 complexes predicted by ClusPro [17] contained the antiparallel α1-α3-α4 arrangment, and all of the top 20 predicted complexes utilized the α1-α3-α4 surface of IPS1 (Fig 2A). Using Dot2 [18] as an alternative docking protocol, all of the top 5 RIG-I—IPS1 complexes had the antiparallel α1-α3-α4 interface, as did 9 of the top 10 and 19 of the top 20 (Fig 2B). Manual examination of the antiparallel α1-α3-α4 RIG-I—IPS1 complex suggests a high degree of shape and electrostatic complementarity (Fig 2C), which has been observed for other CARD-CARD complexes [19].

We identified a pair of residue clusters (RIG-I CARD1 Glu66,67 and IPS1 Arg66,67) that were predicted to interact in the RIG-I—IPS1 interface and were located on a loop between helices 4 and 5, minimizing the potential impact of mutations at these residues on overall protein stability and structure (Fig 2C). Mutating RIG-I CARD1 Glu66,67 to Arg disrupted the protein’s binding to wild-type IPS1 (10-fold increase in Kd, 6.9-fold increase in Km, *p*<0.033) (Fig 2D and S6 Fig). Mutating IPS1 Arg66,67 to Glu had an even larger effect on RIG-I—IPS1 binding, increasing Kd by 34.6-fold and Km by 43.8-fold (*p*<0.004) (Fig 2D and S6 Fig). Similar mutations did not strongly affect RIG-I—IPS1 interactions in the presence of ubiquitin chains [16], suggesting that ubiquitin likely alters the RIG-I—IPS1 interface.

The MDA5-IPS1 complex was less consistently recovered. Although the top-scoring ClusPro complex had the same antiparallel α1-α3-α4 interface that we observed for RIG-I, only 3 of the top 5 (7 of the top 10) coplexes had this arrangement (S7 Fig). MDA5-IPS1 docking using the alternative Dot2 protocol only recovered a similar antiparallel α1-α3-α4 interface in 12 of the top 20 complexes (S7 Fig). We therefore consider prediction of the MDA5-IPS1 complex ambiguous.

Protein-protein docking is a challenging problem, and error rates have historically been notoriously high [20]. However, recent advances have made dramatic improvements in accuracy, particularly for cases such as RIG-I—IPS1 binding, in which the unbound and bound structures are expected to be highly similar [13, 16, 21]. Although the results of mutation experiments were consistent with the predicted antiparallel α1-α3-α4 RIG-I—IPS1 complex, detailed structural studies will be required to determine the precise RIG-I—IPS1 interface when ubiquitin is not present.

### RLR and IPS1 CARD Domains Proliferated in Early Deuterostomes

**Fig 3:**
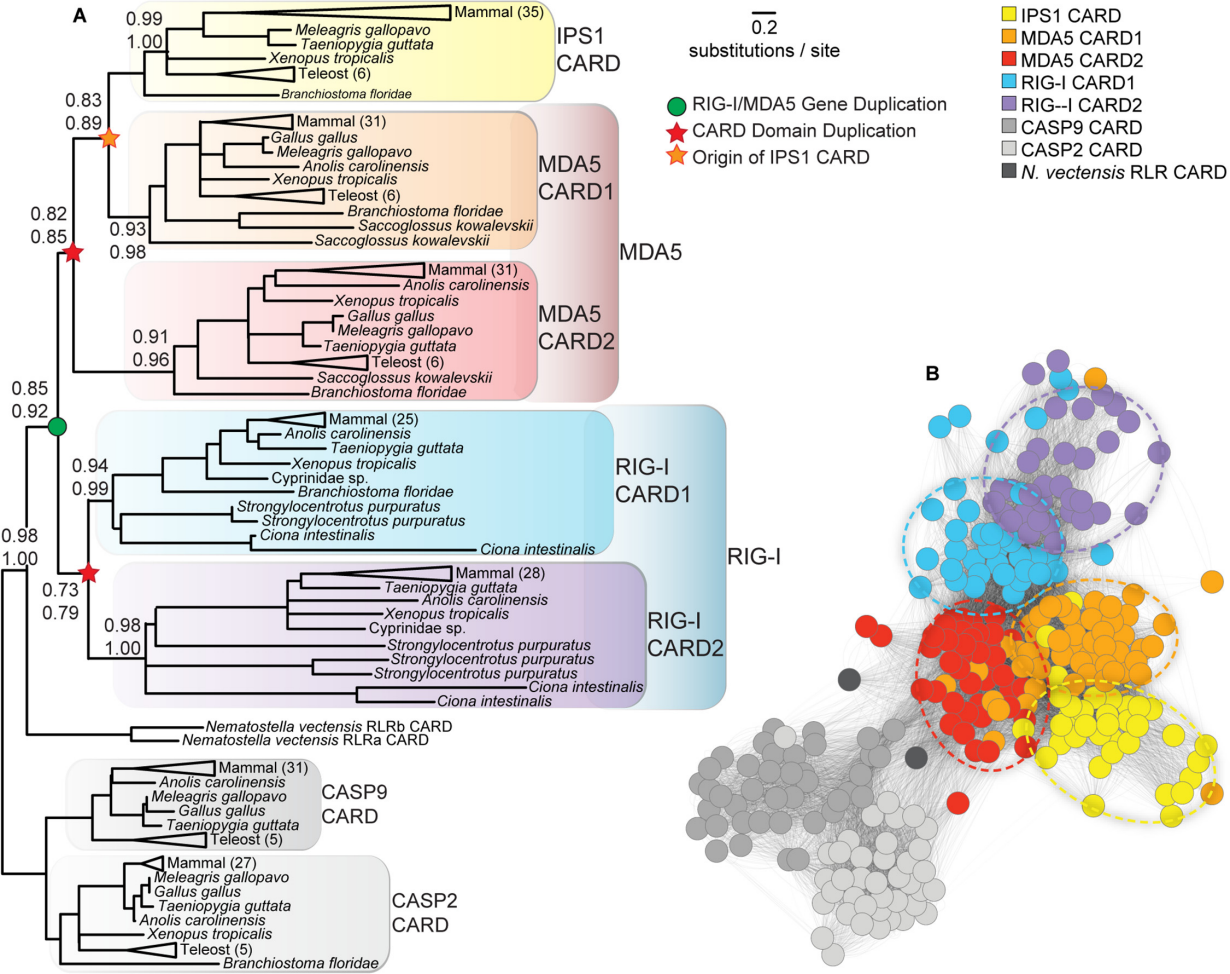
RIG-like receptor (RLR) and IPS1 CARD domains diversified in early deuterostomes. (A) We inferred the consensus phylogeny of RLR and IPS1 CARD domains using maximum likelihood and Bayesian inference (see Materials and Methods). We report statistical confidence supporting key nodes on the phylogeny using maximum-likelihood SH-like aLRT (top) and Bayesian posterior probability (bottom); branch lengths are scaled to substitutions/site. Inferred gene and CARD duplication events are indicated by circles and stars, respectively. (B) We calculated BLAST e-values between all pairs of RLR and IPS1 CARD protein sequences and visualized sequence similarity using a force-directed network layout implemented in Cytoscape v2.8 [22, 23]. Nodes in the network represent individual sequences and are colored by CARD type. Edges indicate pairwise e-value calculations; e-values > 10^−3^ are not shown, to improve clarity. Nodes close to one another are more similar, based on e-value calculations, than nodes far away from one another. The central cluster of each RLR and IPS1 CARD domain is indicated by a dotted oval.

We inferred the phylogeny of RLR and IPS1 CARD domains by maximum likelihood (ML) and Bayesian analyses, using CARD sequences from caspases 9 and 2 as outgroups. The resulting consensus tree (Fig 3A) recovered individual deuterostome IPS1, MDA5 CARD1, MDA5 CARD2, RIG-I CARD1 and RIG-I CARD2 clades with > 90% ML SH-like aLRT and > 95% Bayesian posterior probability, suggesting that RLR and IPS1 CARD domains diversified in deuterostomes before the vertebrate lineage. Both the ML and Bayesian phylogenies inferred that RIG-I and MDA5 CARD domains duplicated independently following the RIG-I/MDA5 gene duplication, suggesting that the CARD1+CARD2 domain architecture arose independently in RIG-I and MDA5, with IPS1 originating from a later duplication of MDA5 CARD1. The alternative phylogeny, in which CARD domain duplication precedes the RIG-I/MDA5 gene duplication (S8 Fig), was rejected as a plausible explanation of our sequence data, with marginal significance (SH-test *p* = 0.02). However, the statistical support in favor of the maximum-likelihood branching pattern is weak. Removal of fast-evolving non-vertebrate sequences improved inferred support for the consensus branching pattern but did not fully resolve the tree (S9 Fig).

RLR and IPS1 CARD domains are small and fast-evolving, making it difficult to resolve the branching pattern with high confidence using traditional techniques. We therefore used a variety of alternative approaches to more thoroughly assess the reliability of our phylogenetic inference. First, we used a sequence-similarity network to cluster RLR and IPS1 CARD domains independent of phylogenetic inference [22, 23]. The results of this analysis are largely consistent with the consensus phylogeny (Fig 3B). The caspase 9–2 sequences form a well-defined group that is highly diverged from RLR and IPS1 CARDs, with *Nematostella* RLR CARDs falling between the caspases and the other RLR-IPS1s. The RIG-I CARD domains cluster together in the network, and the MDA5 CARDs form a tight cluster with IPS1 CARD domains, reinforcing the branching pattern observed from phylogenetic analysis.

Second, we constructed hidden Markov models (HMMs) from independent alignments of deuterostome RIG-I—CARD1, RIG-I—CARD2, MDA5-CARD1, MDA5-CARD2 and IPS1-CARD sequences [24, 25], each of which formed a monophyletic clade in the consensus tree with strong support (Fig 3A). We calculated pairwise distances between the HMMs and inferred the neighbor-joining tree from these distances [26, 27], which recovered the same branching pattern as the ML phylogeny based on protein sequence data (S10 Fig). Although these analyses cannot completely resolve the branching pattern explaining RLR-IPS1 CARD proliferation, that the same branching pattern is consistently recovered using a variety of different inference methodologies argues against strong methodological bias.

Although caspase outgroups rooted our RLR-IPS1 CARD phylogeny with strong support, the large evolutionary distance between caspase and RLR-IPS1 sequences invokes the possible specter of long-branch attraction. To address this possibility, we used gene/species tree reconciliation to identify the most likely RLR-IPS1 rooting in the absence of outgroup data [28–30]. The resulting rooting is consistent with that obtained using caspase outgroups (S11 Fig). Although this analysis cannot completely rule out long-branch attraction, it does argue against such an interpretation.

It has been suggested that gene conversion events can homogenize the sequences of duplicate genes, potentially misleading evolutionary analyses [31]. We used pairwise comparisons of closely-related mammalian RLR and IPS1 genes to investigate the extent of gene conversion among RLR and IPS1 CARD domains [32]. We found very little evidence for RLR-IPS1 gene conversion (S1 Table). Only two regions of mammalian MDA5 CARD2 were identified as having undergone potential gene conversion events (*p*<0.03), and neither of these was significant after Bonferroni correction for multiple tests (*p*>0.4).

We conclude that the lack of phylogenetic resolution in the RLR-IPS1 CARD tree likely reflects the ambiguous information present in available sequence data due to fast-evolving, short sequences. Although we could not detect any evidence of methodological bias, we remain cautious in our interpretation of the precise branching pattern explaining the diversification of RLR and IPS1 CARD domains. However, the conclusion that RLR-IPS1 CARDs proliferated in deuterostomes prior to the vertebrate origin appears robust.

### RLR and IPS1 Genes Are Tightly Clustered in *Branchiostoma floridae*

Our phylogenetic analysis suggests that RLR and IPS1 CARD domains proliferated in early deuterostomes (Fig 3). Insight into a plausible mechanism for generating this series of partial-gene duplications comes from examining where RLR and IPS1 genes are located in the *Branchiostoma floridae* genome, a cephalochordate that diverged from other deuterostomes shortly before the vertebrate lineage [33].

**Fig 4:**
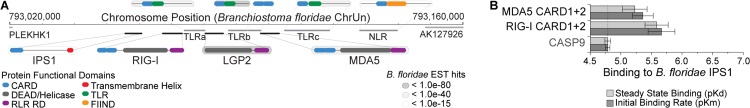
Deuterostome RIG-like receptors (RLRs) and IPS1 cluster with other potential CARD-signaling immune receptors in the *Branchiostoma floridae* genome. (A) We plot the physical location of each identified RLR and IPS1 gene along the assembled *B*. *floridae* chromosome (bottom). Other genes in the cluster are shown along the top. Functional domains are indicated by colors. Genes with significant BLAST hits to the *B*. *floridae* expressed sequence tag (EST) database are shaded according to e-value. (B) We measured the kinetics of *B*. *floridae* RIG-I CARD1+2 and MDA5 CARD1+2 domains binding to *B*. *floridae* IPS1 CARD in vitro (see Materials and Methods) (S1 Fig). We plot the mean and standard error of–log-transformed steady-state dissociation (pKd) and initial binding rate (pKm), with human CASP9 used as a negative control. Dotted vertical line indicates approximate level of non-specific binding, with longer bars indicating tighter binding (S12 Fig).

We found that all three RLRs (RIG-I, MDA5 and LGP2), as well as IPS1, three possible toll-like receptors and one possible nod-like receptor are all located within a short 100-kb region of the assembled *B*. *floridae* chromosome (Fig 4A). Inferred coding sequences encoded proteins having domain architectures equivalent to those found in human proteins, including the twin N-terminal CARD domains of RIG-I and MDA5. The *B*. *floridae* IPS1 gene encoded an N-terminal CARD domain as well as an unstructured, proline-rich region and a predicted C-terminal transmembrane helix, all of which is characteristic of vertebrate IPS1. We found near-exact matches to some of our inferred RLR genes in expressed sequence tag (EST) databases, arguing that these genes are likely functional in *B*. *floridae* and not pseudogenes or gene modeling errors (Fig 4A).

We measured the binding of *B*. *floridae* RIG-I CARD1+2 and MDA5 CARD1+2 domains to the *B*. *floridae* IPS1 CARD domain and found that RIG-I bound IPS1 significantly more tightly than a negative control (.5.4-fold lower Kd, 6.1-fold lower Km, *p*<0.044) (Fig 4B and S12 Fig). The difference in binding of *B*. *floridae* MDA5 CARD1+2 vs. the negative control was not significant (*p*>0.07). However, these results do suggest that *B*. *floridae* has functional RLR genes that are tightly clustered in its genome—along with IPS1 and other immune-system genes—and at least some of these RLRs are likely to signal through IPS1.

Interestingly, all the genes present in this cluster encode proteins with likely immune-signaling functions, and all of them contain homologous CARD domains expected to be involved in CARD-CARD signaling interactions. This suggests a plausible mechanism by which a series of tandem, partial-gene duplication events may have given rise to the diversity of RLR-IPS1 CARD sequences observed in deuterostomes. A dual-CARD open reading frame just upstream of *B*. *floridae* MDA5 that is 98% identical to the MDA5 CARD1+2 protein sequence further reinforces this conclusion and suggests that tandem partial-gene duplications may be ongoing.

Although most of the sequenced genomes of other pre-vertebrate deuterostomes have not been assembled to chromosomes, we did observe some evidence for possible genomic clustering of at least some of the RLRs in *Ciona intestinalis* and other deuterostome genomic scaffolds (S13 Fig). Additionally, recombination is expected to break down gene clusters over large evolutionary timescales, whereas the de novo generation of functionally-linked gene clusters is much less likely [34]. Together, these results suggest that RLRs and IPS1 were probably clustered in the ancestral deuterostome.

Tandem gene and partial-gene duplications—facilitated by unequal crossing over and other mechanisms—have been widely observed in animal and plant genomes [35]. Our results suggest that the tight clustering of RLR and IPS1 genes in the ancestral deuterostome genome may have facilitated CARD domain proliferation by bringing similar sequences into close physical proximity, allowing unequal crossing over or other mechanisms to generate multiple rounds of tandem partial-gene duplications.

### Coevolution between RLRs and IPS1 Differentiated Early Deuterostome RLR-IPS1 Signaling Interactions

Examination of RLR-IPS1 binding in pre-vertebrate deuterostomes can suggest possible ancestral molecular functions but cannot explicitly test these hypotheses. To directly measure the interactions of early deuterostome proteins, we reconstructed ancestral sequences (S1 Fig) using phylogenetic techniques that explicitly incorporate uncertainty about the tree topology (see Materials and Methods). Consistent with previous findings that ancestral reconstruction is highly robust to phylogenetic uncertainty [36], we found that ancestral deuterostome RLR and IPS1 CARD domains were reconstructed with high confidence, even though the tree topology was ambiguous (S14 Fig). Across all ancestral sequence reconstructions, over 55% of residues were reconstructed with posterior probability > 0.95; over 60% were reconstructed with posterior probability > 0.90, and over 70% were reconstructed with posterior probability > 0.80. Furthermore, only 91/960 (<9.5%) reconstructed positions had an alternative reconstruction with posterior probability > 0.3, and the majority of these (93%) were in the same general biochemical class as the maximum-likelihood reconstructed residue. Together, these results suggest that ancestral sequences were reconstructed with high confidence and low ambiguity, despite the ambiguity in the underlying phylogeny.

High statistical confidence in reconstructed ancestral sequences does not necessarily imply that the sequences are accurate. To better characterize the expected error rates of reconstructed ancestral sequences, we simulated protein sequence data along the maximum-likelihood phylogeny using the best-fit evolutionary model and ML parameter estimates. We found that ancestral reconstruction error rates under this scenario were < 0.05 for all nodes examined in this study (< 6 errors/sequence) (S14 Fig). When amino-acid residues with similar biochemical properties were considered equivalent, error rates were reduced to < 0.02 (S14 Fig).

When the assumed evolutionary model differs markedly from the actual molecular-evolutionary process, phylogenetic analyses such as ancestral sequence reconstruction can produce unreliable results. Recent studies have suggested that changes in state frequencies across the phylogeny can lead to errors in ancestral sequence reconstruction [37]. Although we did observe differences in amino-acid residue patterns across RLR and IPS1 CARD sequences (S10 Fig), none of the amino-acid frequency distributions differed significantly from the frequency distribution across all sequences (chi-squared *p*>0.74), suggesting that amino-acid frequencies are roughly stationary across the tree (S14 Fig). Although it is impossible to completely rule out errors due to model misspecification, we observed no evidence for strong model violations or other artifacts that might affect ancestral sequence reconstruction.

**Fig 5:**
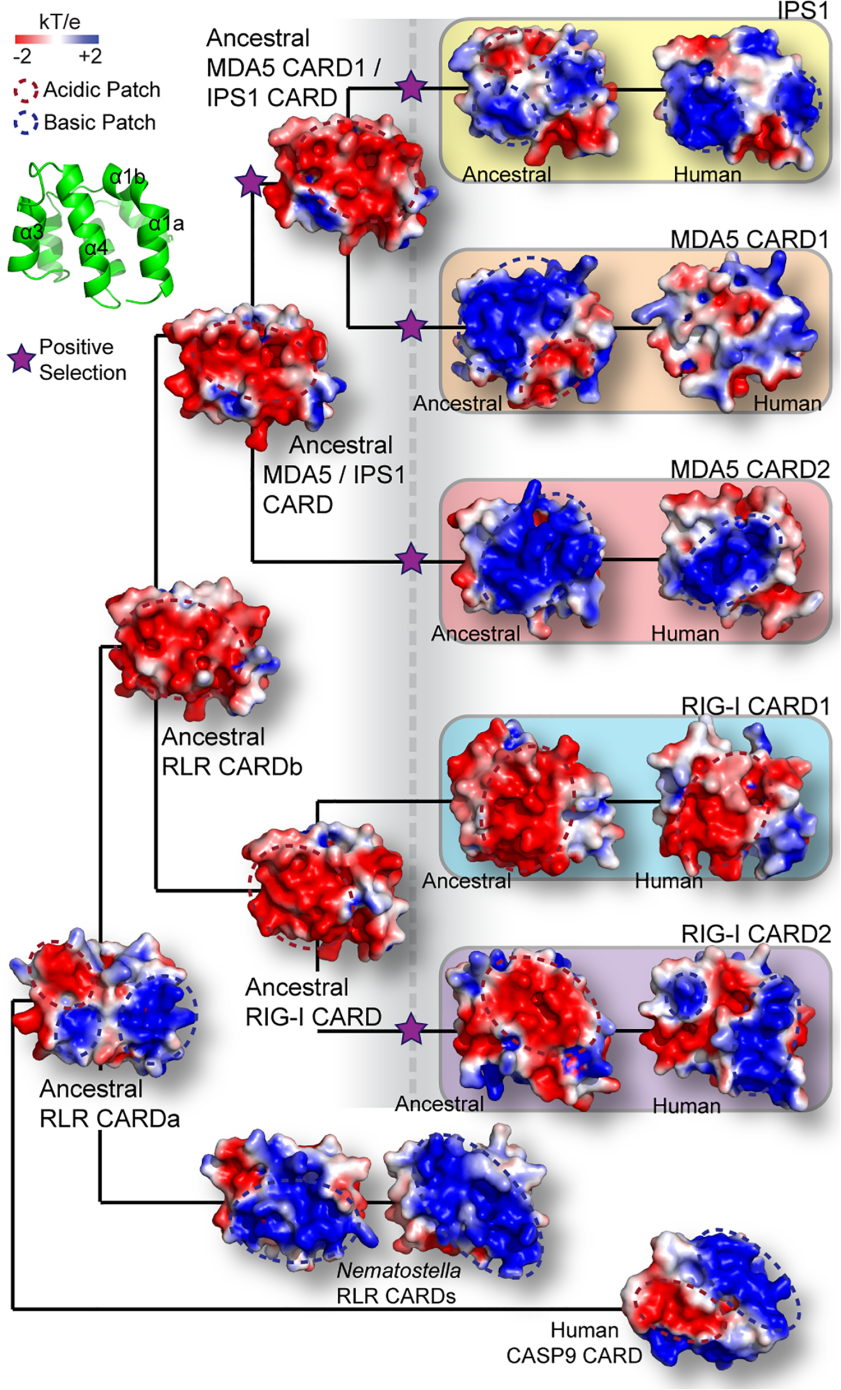
RIG-like receptor (RLR) and IPS1 CARD domains structurally diverged in early deuterostomes, and this structural divergence was associated with protein-coding adaptation. We inferred the 3-dimensional structures of ancestral RLR and IPS1 CARD domains at key points on the RLR-IPS1 CARD phylogeny (see Materials and Methods) (Fig 3). We plot the electrostatic surface potential (kT/e) across the α1-α3-α4 surface of each CARD domain, with large acidic and basic patches indicated by dotted red and blue ovals, respectively. We additionally used phylogenetic techniques to infer the presence of adaptive protein-coding substitutions on each branch of the phylogeny (see Materials and Methods). Stars indicate significant support for protein-coding adaptation on the indicated branch (*p*<0.05 after correcting for multiple tests). Large dotted vertical line indicates the approximate time of RLR-IPS1 CARD proliferation in early deuterostomes.

Structural modeling of ancestral RLR and IPS1 CARD domains identified large changes in electrostatic charge across the surfaces of these molecules (Fig 5). Interestingly, major evolutionary shifts in electrostatic charge distribution across ancestral CARD domains coincided with inferences of significant protein-coding adaptation on those branches of the phylogeny, suggesting a model in which positive selection may have driven structural changes in RLR and IPS1 CARDs early in deuterostome evolution (Fig 5). Specifically, the predicted α1-α3-α4 RLR-IPS1 interaction interface was inferred as strongly acidic in all ancestral CARD domains before the deuterostome proliferation (ancRLR CARDb, ancRIG-I CARD, ancMDA5/IPS1 CARD, and ancMDA5 CARD1 / IPS1 CARD) (Fig 5). This acidic α1-α3-α4 surface remained largely acidic in the ancestral and human RIG-I CARD1 domains but appears to have acquired large basic patches in other RLR and IPS1 CARDs, after the deuterostome proliferation (boxed ancestral and human CARDs) (Fig 5). On every branch across which we observed the acquisition of basic patches on the α1-α3-α4 surface, we also inferred protein-coding adaptation by nonsynonymous/synonymous substitution analysis (see Materials and Methods) (Fig 5). That the distribution of basic patches across the α1-α3-α4 surface differs markedly among different CARD domains reinforces the phylogenetic conclusion that these basic patches were independently acquired.

Although nonsynonymous/synonymous substitution comparisons are widely used to characterize protein-coding adaptation and are generally considered robust [38–41], concerns have been raised that such analyses may suffer from high false-positive error rates under some conditions [42, 43]. We examined potential false-positive error rates by simulating codon sequence data along the maximum-likelihood RLR-IPS1 CARD phylogeny under three different empirically-derived evolutionary models (S15 Fig). False-positive error rates were highest when sequences evolved neutrally across the entire tree but were never greater than 0.05. Error rates were lower (mean = 0.009, SE = 0.0044) when sequences evolved under an empirical model with all parameters estimated from our sequence data and intermediate (mean = 0.024, SE = 0.006) when sequences evolved under an empirical model but were released from selective constraint on the branch being tested. That false-positive error rates were low across a variety of scenarios suggests that our analysis of protein-coding adaptation is likely reliable, although it is impossible to completely rule out potential model violations.

As CARD-CARD signaling interactions are known to be facilitated by surface charge complementarity [19], we hypothesized that the structural changes we observed were responsible for establishing the specific CARD-CARD interactions facilitating ancestral RLR-IPS1 binding in deuterostomes.

To examine this hypothesis, we expressed and purified ancestral RLR and IPS1 CARD domains and measured their binding affinities in vitro. Binding to human caspase 9 CARD was used as a negative control. Examining the initial establishment of RLR-IPS1 CARD-CARD interactions is complicated, as IPS1 CARD appears to have arisen via the duplication of MDA5 CARD1 into a new gene context, and the precise branching pattern by which RLR-IPS1 CARD domains proliferated cannot be unambiguously determined (Figs 3 and 5). We focused on characterizing the potential ancestral RLR-IPS1 CARD-CARD interactions before the deuterostome proliferation (dotted vertical line) (Fig 5), at which time each RLR had a single CARD domain (ancRIG-I CARD and ancMDA5/IPS1 CARD) (Fig 5), and the ancestral MDA5 CARD1 / IPS1 CARD was present as a potential interaction partner. We compared results using these pre-deuterosome-proliferation sequences to results obtained using ancestral RLR and IPS1 CARD sequences reconstructed after the deuterosome proliferation to determine how initial RLR-IPS1 CARD-CARD interactions may have established themselves as these domains proliferated.

**Fig 6:**
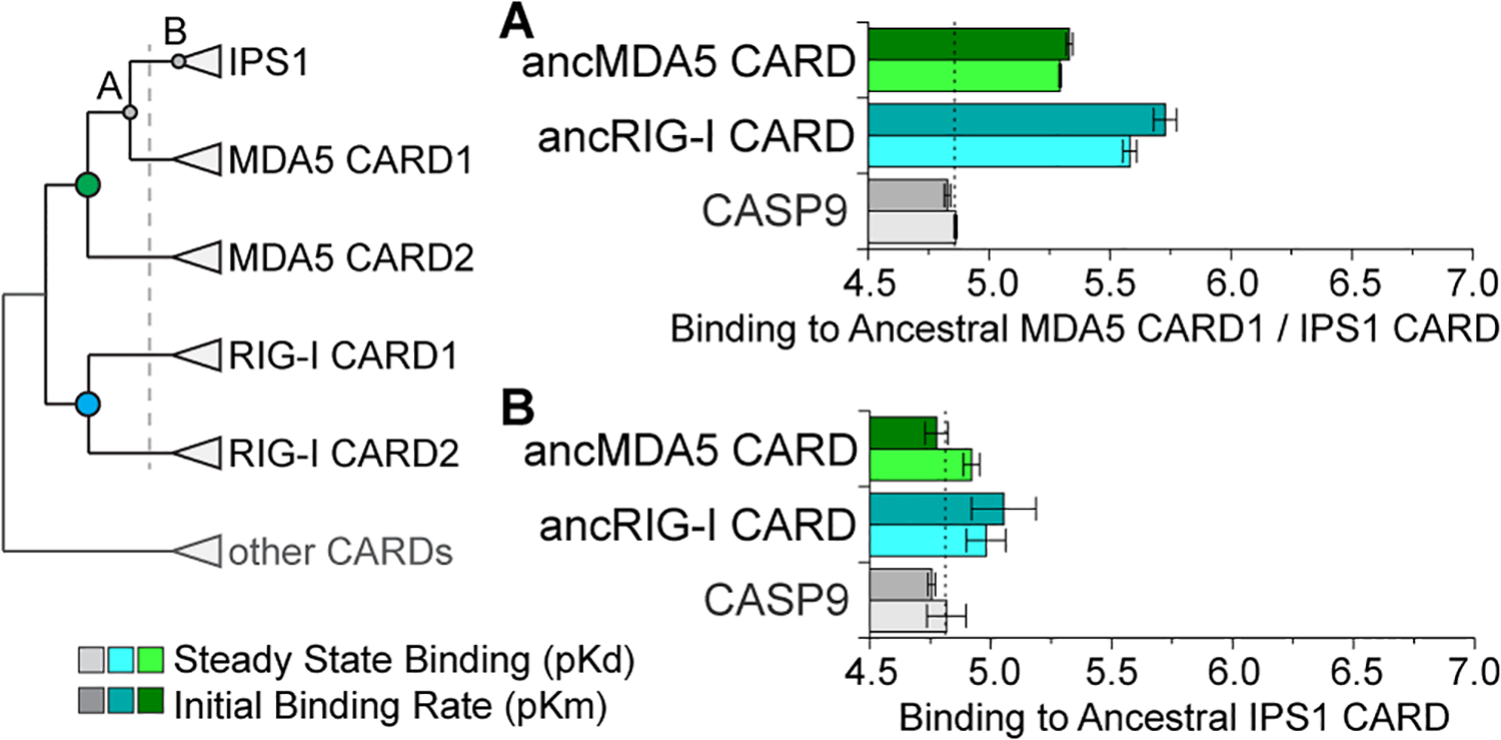
Ancestral RIG-like receptor (RLR) CARDs bound the IPS1 CARD progenitor before the proliferation of RLR-IPS1 CARD domains in early deuterostomes, but did not bind the ancestral IPS1 CARD after it diverged from RLR CARDs. We resurrected ancestral RLR and IPS1 CARD domains and measured their binding kinetics in vitro (see Materials and Methods) (S1 Fig). The simplified phylogeny at left indicates the ancestral proteins examined in this set of experiments, with the dotted vertical line indicating the approximate time of the RLR-IPS1 CARD proliferation in deuterostomes (Figs 3 and 5). Colored circles indicate ancestral RLR CARDs, while gray circles indicate the ancestral IPS1 CARD progenitor before (A) and after (B) the deuterostome proliferation. We show the mean and standard error in–log-transformed steady-state dissociation (pKd) and initial binding rate (pKm) of in vitro kinetics assays, with dotted vertical line indicating approximate nonspecific binding (indicated by CASP9 negative control) and longer bars indicating tighter binding (S16 Fig).

We found that, prior to the deuterostome proliferation, ancestral RIG-I and MDA5 CARD domains (ancRIG-I CARD and ancMDA5/IPS1 CARD) (Fig 5) bound the ancestral MDA5 CARD1 / IPS1 CARD with high affinity (*p*<0.0016) (Fig 6A and S16 Fig), suggesting that, prior to RLR-IPS1 CARD proliferation, the potential for an ancestral RLR CARD–IPS1 CARD interaction existed. Partial-gene duplication of MDA5 CARD1 into an IPS1-like architecture could have recruited this potential to establish a novel protein-protein signaling interaction without requiring any changes in protein sequence.

However, neither of the ancestral RLR CARDs before the deuterostome proliferation bound the ancestral IPS1 CARD after it diverged from MDA5 CARD1 (ancIPS1 CARD) (Fig 5), suggesting that, after the divergence of IPS1 from the RLRs, substitutions accrued in IPS1 CARD that prevented it from binding to its potential ancestral signaling partners (*p*>0.15) (Fig 6B and S16 Fig).

**Fig 7:**
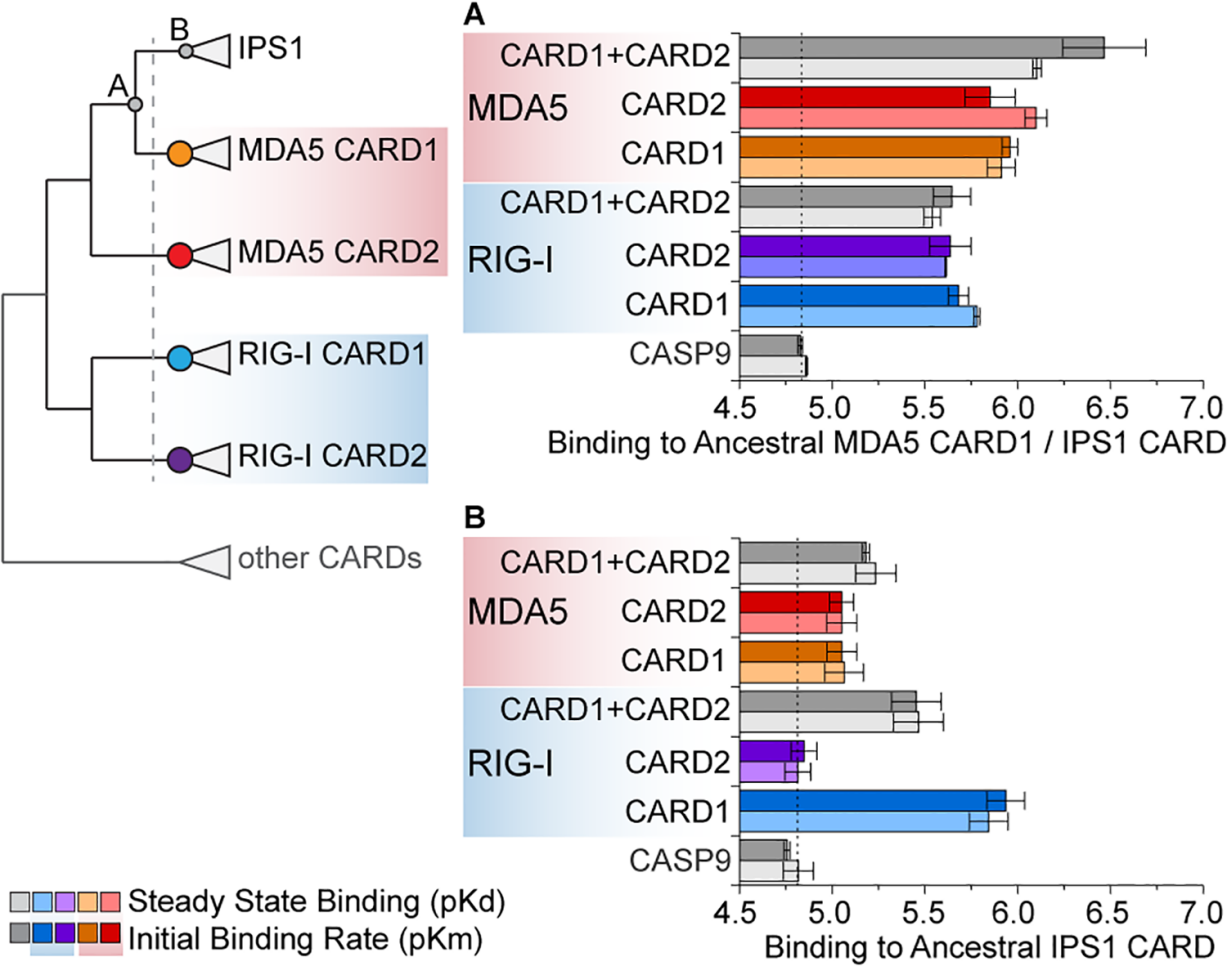
Functional differentiation of ancestral RIG-I CARD1 and CARD2 domains occurred after the proliferation of RLR-IPS1 CARDs in deuterostomes, due to coevolution between RIG-I and IPS1 CARDs. Ancestral RLR and IPS1 CARD domains were resurrected, and we measured the kinetics of RIG-I and MDA5 CARDs bound to IPS1 CARD before (A) and after (B) the RLR-IPS1 CARD proliferation in early deuterostomes (dotted vertical line on simplified CARD phylogeny) (Figs 3 and 5). We plot the mean and standard error of–log-transformed steady-state dissociation (pKd) and initial binding rate (pKm) when ancestral RLR CARD domains bind to IPS1 CARD before (A) and after (B) the deuterostome proliferation (see Materials and Methods). The approximate level of nonspecific binding is indicated by a vertical dotted line (inferred using human CASP9), and longer bars indicate tighter binding (S17 and S18 Figs).

After the deuterostome proliferation, we found that compensatory substitutions occurred in RIG-I and MDA5 CARD domains that differentially re-established their ability to bind the diverged IPS1 CARD (Fig 7). Interestingly, all the diverged RIG-I and MDA5 CARD domains bound the ancestral MDA5 CARD1 / IPS1 CARD better than the negative control, suggesting that compensatory substitutions in RLR CARD domains did not interfere with their ability to bind their potential pre-deuterostome-proliferation partner (*p*<0.018) (Fig 7A and S17 Fig). However, after the deuterostome proliferation, only the ancestral RIG-I CARD1 and combined MDA5 CARD1+2 bound the diverged IPS1 CARD better than the control (*p*<0.003 for RIG-I CARD1, *p*<0.04 for MDA5 CARD1+2) (Fig 7B and S18 Fig).

In the case of the ancestral deuterostome RIG-I, we observed the same functional differentiation that we observed in human RIG-I (Fig 1B). The ancestral RIG-I CARD1 bound ancestral IPS1 CARD (Kd = 5.89e^-6^ M, Km = 1.15e^-6^ M, *p*<0.003); ancRIG-I CARD2 did not (*p>*0.31), and the combined RIG-I CARD1+2 domain bound similarly to CARD1, alone (*p*>0.09) (Fig 7B and S18 Fig). In contrast, although the ancestral MDA5 CARD1+2 bound ancestral IPS1 CARD (Kd = 5.89e^-6^ M, Km = 6.61e^-6^ M, *p*<0.04), ancestral MDA5 CARD1 and ancMDA5 CARD2, alone, were not significantly better than the negative control (*p*>0.06) (Fig 7B and S18 Fig), suggesting that the similar functional differentiation of MDA5 CARD1 and CARD2 that we observed in human MDA5 (Fig 1B) must have occurred later in the MDA5 lineage.

Together, these results suggest that early coevolution between RLRs and IPS1 was responsible for differentiating RLR-IPS1 CARD-CARD interactions in deuterostomes. Before the proliferation of RLR and IPS1 CARDs in deuterostomes, both RIG-I and MDA5 CARD domains had the potential to bind an IPS1-like signaling partner (Fig 6A). After the deuterostome divergence, all RLR CARDs retained their capacity to bind the ancestral MDA5 CARD1 / IPS1 CARD (Fig 7A), but only RIG-I CARD1 and the combined MDA5 CARD1+2 could bind the diverged IPS1 (Fig 7B). The changes that accrued in IPS1 CARD as it diverged from the RLR CARD domains also interfered with its capacity to bind its potential ancestral partners (Fig 6A), suggesting that coevolution between IPS1 and RLR CARD domains was responsible for differentiating RLR-IPS1 interactions. Whether molecular adaptation drove the establishment of these interactions or was a result of selection for other functions is unclear. The implications of these molecular interactions for the evolution of cellular immune signaling are also unclear, as interactions with ubiquitinating enzymes and binding of free polyubiquitin could also have regulated how direct RLR-IPS1 CARD-CARD interactions functioned in early cellular immunity.

### Conclusion

The importance of the RIG-like receptors (RIG-I, MDA5 and LGP2) to antiviral immunity is well established, but we still lack a precise understanding of the specific biochemical interactions responsible for RLR-based immune signaling. An emerging picture—based on detailed analyses of human and other vertebrate RLRs—suggests that RIG-I and MDA5 activate immune responses primarily through direct interactions with IPS1, mediated by homologous RLR and IPS1 CARD domains and modulated by K63-linked polyubiquitin chains and ubiquitinating enzymes [5, 6, 14, 15].

Although recent studies have highlighted the importance of ubiquitinating enzymes and binding of free polyubiquitin chains for RLR-based immune signaling [14, 15], the mechanisms by which ubiquitin may facilitate immune activation remain unclear. Ubiquitin chains might enhance RLR binding to specific signaling partners, facilitate localization of RLRs to the mitochondrial membrane, generate or stabilize oligomeric signaling complexes, occlude binding sites used by negative regulators or function through other mechanisms.

Structural and mutational studies have suggested that—in the presence of free K63-linked polyubiquitin—it is the CARD2 domain of RIG-I that directly binds IPS1 CARD [13, 16]. However, here we demonstrate that—in the absence of ubiquitin—RIG-I and MDA5 CARD1 domains are primarily responsible for mediating direct binding to IPS1 CARD. Our results do not contradict the body of structural and mutational work showing that RIG-I CARD2 interacts directly with IPS1 in the presence of free ubiquitin. Rather, we feel our results suggest that free ubiquitin may modulate the RLR-IPS1 interface. Covalently linked polyubiquitin may further modulate this interface, although further studies will be required to examine these hypotheses.

Our evolutionary analyses characterize how the RLR-IPS1 interaction—absent ubiquitin—changed over time. The consequences of these changes in RLR molecular function for the evolution of cellular immune signaling remain unclear, as free ubiquitin and ubiquitinating enzymes are likely to have played a role in the evolution of RLR-IPS1 immune signaling. Larger evolutionary studies examining the complete RLR-IPS1-ubiquitin signaling system will be required to understand how the evolution of specific molecular-functional aspects of this system impacted organismal evolution.

In contrast to the relatively large body of work examining RLR-IPS1 interactions in mammals and other vertebrates, very little is known about how RLRs and IPS1 evolved. Earlier studies suggested that RLRs arose and diversified in vertebrates through complex evolutionary processes [44, 45]. In our view, these early studies suffered from incomplete sequence data. Our previous work and this study argue that RLRs arose in early metazoa and diversified through a series of gene duplication events [10]. Our phylogenetic analyses—which appear robust to major error—further suggest that IPS1 arose much later than the RLRs, probably in early deuterostomes. This finding raises the question, how did the RLRs function before the origin of their canonical signaling partner? Examination of RLR-based immune signaling in early animals will likely be required to answer this question and is expected to further illuminate the mechanisms driving the evolution of animal innate immunity.

**Fig 8:**
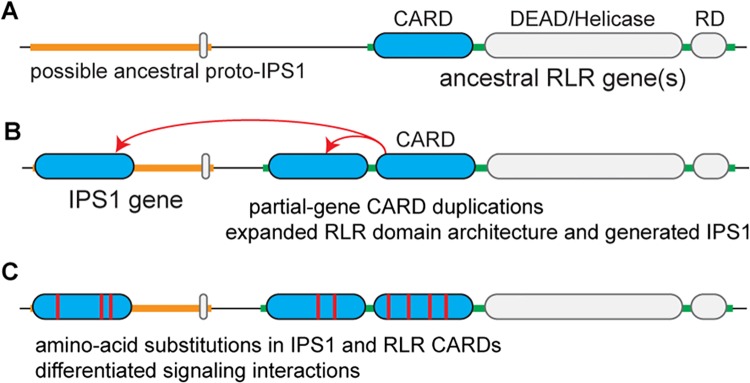
Our results suggest a model in which partial-gene duplication and subsequent coevolution generated RLR-IPS1 interactions. (A) Prior to the deuterostome proliferation of RLR and IPS1 CARD domains, ancestral RLR(s) functioned through an unknown immune-signaling mechanism. (B) Partial-gene duplications of RLR CARD(s) in deuterostomes generated the canonical RIG-I—MDA5 2-CARD domain architecture as well as the IPS1 gene, creating a new potential immune-signaling partnership. (C) Coevolution between RLR and IPS1 CARD domains differentiated RLR-IPS1 signaling interactions. Note that this model is parsimonious, in that it only posits the necessary duplications of CARD signaling domains. More complex scenarios are also possible, such as the complete duplication of one of the RLRs, followed by loss of RNA-binding domains to produce IPS1. Whether RLR-IPS1 evolution followed our simple parsimonious model or a more complicated scenario cannot be determined, given current data.

How novel protein-protein signaling interactions arise and establish themselves as distinct from existing interactions has been a long-standing question in molecular evolution, but we have had very little direct information to address this question. Our examination of RLR-IPS1 interactions across evolutionary history suggests a general model in which the duplication of individual domains involved in existing protein-protein interactions into new gene contexts could facilitate the immediate establishment of new functional interactions (Fig 8A and 8B). Subsequent coevolution between these new interaction partners could then differentiate the newly-established signaling interaction from other existing interactions (Fig 8C). Our work also suggests that gene clusters may play an important role in facilitating the generation of novel protein-protein interactions by bringing different genes into close physical proximity, allowing unequal crossing-over and other localized processes to duplicate functional domains into new gene contexts.

## Materials and Methods

### Binding Kinetics Analysis

CARD proteins were expressed in *E*. *coli* Rosetta 2(DE3)pLysS cells using pET-22b(+) constructs, which were verified by Sanger sequencing (S1 Fig). 500-mL cultures were inoculated with 10-mL suspended cells and incubated for 3 hours at 37º C. Cells were lysed using mechanical disruption with MP Biomedicals Fastprep 24, and cellular material was filtered by centrifugation. CARD proteins were purified under non-denaturing conditions by His-affinity purification using an elution buffer containing 50 mM sodium phosphate, 300 mM sodium chloride and 150 mM imidazole at pH 7.4. Resulting purified protein was dialyzed overnight at 4º C in 1000 mL PBS buffer using a 3500 MWCO dialysis membrane to remove salts and other unwanted components. Proteins were visualized by SDS-page stained with 1% coomassie to confirm purity, and protein concentrations were measured via a linear-transformed Bradford assay [46]. IPS1 CARDs were FLAG-tagged on the N-terminus to facilitate binding kinetics assays. RLR CARDs were not FLAG-tagged.

We measured IPS1-RLR CARD-CARD binding using a label-free in vitro kinetics assay at pH 7.0 [47]. IPS1 CARD proteins at 2.5 μg/mL concentration were immobilized to a series of 8 anti-FLAG probes for 5 minutes, until saturation was observed. Probes were washed in 1x Kinetics Buffer (ForteBio) and then exposed in parallel to RLR CARDs at 0.001–100 μM concentrations in 1x Kinetics Buffer for 60 minutes, at which point binding curve saturation was observed, and all probes were allowed to dissociate in Kinetics Buffer for an additional 60 minutes. All kinetics buffers included BSA to control for non-specific binding. CARD-CARD association and dissociation were measured as the nanometer shift in laser wavelength reflected through each probe over time, sampled every 3 milliseconds. Results from a parallel probe run at 0 μM RLR CARD concentration were subtracted to account for system fluctuation over time.

Steady-state molecular binding at saturation was measured as the change in laser wavelength averaged over the last 500 samples during CARD-CARD association. We additionally fit association-dissociation curves to the kinetics data in order to estimate the initial rates of CARD-CARD binding. Measurements were taken from 3 experimental replicates, each of which produced 7 normalized association-dissociation plots across RLR CARD concentrations.

We estimated RLR CARD concentrations at which ½-maximal steady-state CARD-CARD binding (Kd) and ½-maximal initial binding rate (Km) were achieved using two different approaches, which produced qualitatively equivalent results. First, we estimated Kd and Km values separately using the data generated from each experimental replicate and then calculated average Kd and Km values across replicates. Second, we calculated the average and standard-error of each of the 7 concentration-response data points across the 3 experimental replicates and estimated Kd and Km values from these ‘average’ data. Results from the first approach are presented as bar plots and discussed in the main text. Results from the second approach are displayed as concentration-response graphs in supplementary figures. In either case, Kd and Km were estimated by fitting a one-site binding curve using nonlinear regression. All curve fits were inferred with r^2^>0.91. Differences in Kd and Km were assessed using two-tailed, two-sample *t* tests, assuming unequal variances between samples. For visualization, we report–log_10_-transformed Kd (pKd) and Km (pKm).

### Sequence Identification and Alignment

Full-length RLR and IPS1 protein sequences were identified by sequence search of the NCBI RefSeq and Ensembl databases [48, 49], using human RLRs and IPS1 as queries and a BLASTP e-value cutoff of 10^−10^ [50]. Protein sequences from well-curated vertebrate genomes were then used to refine RLR and IPS1 gene models in the genomes of less reliably-curated deuterostomes by homology-based gene identification using FGENSH+ [51].

RLR and IPS1 CARD domains were identified by sequence search of the Pfam database [52], the Conserved Domain Database [53] and the SMART database [54]. Domain boundaries were further refined by aligning RLR and IPS1 sequences to those whose CARD domains have been structurally determined (PDB IDs: 4A2W, 4A2Q, 2LWD, 2LWE, 4NQK, 2VGQ, 3YGS, 3J6J, 4P4H).

CARD domain sequences were aligned using the G-INS-i algorithm in Mafft v6.850b [55] and using Muscle v3.8.31, with default parameters [56]. Alignments were further manually refined by comparing the sequence alignment to a structural alignment of the above PDB IDs, produced using the CEAlign algorithm available in PyMOL v1.7 [57].

### Phylogenetic Analysis

The maximum-likelihood (ML) phylogeny was inferred using PhyML v3.0 [58], with the best-fit evolutionary model selected by AIC in ProtTest v3 [59]. We used the LG substitution model [60], estimating the residue frequencies from the alignment data and allowing for an 8-category discrete-gamma approximation of among-site rate variation. The tree topology was inferred by heuristic search using a combination of SPR and NNI rearrangements. Support for each clade was assessed using SH-like aLRT [61].

Bayesian phylogenetic analysis was conducted using MrBayes v3.2.1 [62, 63]. We executed 2 independent runs, with 4 chains/run, and terminated the analysis when the deviation in average clade posterior probabilities between the 2 runs fell below 0.01. Tree topologies were sampled every 100 steps in the MCMC chains, with the first 25% of samples discarded as burnin. The amino-acid substitution model was integrated over using a mixed prior; the proportion of invariant sites was set to zero, and all other prior distributions were set to default values.

Protein-coding adaptation was inferred using the branch-sites test in PAML v4.7 [39, 64]. Each gene’s codon sequence was mapped to the amino-acid alignment, and the likelihood of the ML tree was calculated under two evolutionary models, one in which an inferred proportion of codons are allowed to have a nonsynonymous/synonymous rate ratio (ω) > 1 on a single branch, indicative of positive selection on the protein sequence, and a null model that constrains ω≤1 for all sites and branches. We calculated the likelihood ratio of the positive-selection model to the null model and assessed significance using a Bonferroni-corrected chi-square test [39].

### Ancestral Sequence Reconstruction

Ancestral protein sequences were reconstructed using the June, 2012 release of Lazarus, which automates the integration of ancestral sequence reconstructions across multiple tree topologies [36]. We included the ML topology, the alternative topology (S8 Fig), and all topologies in the 95% credibility set inferred by Bayesian phylogenetic analysis. Ancestral protein sequences were inferred from each topology by marginal reconstruction using PAML v4.7 [64, 65], assuming the best-fit LG+F+G evolutionary model. The posterior probability of each ancestral-reconstructed residue was inferred by PAML and weighed by the empirical-Bayesian posterior probability of the tree on which it was reconstructed. Equivalent ancestral sequences were obtained using RAxML v8.0.0 [66]. Insertions and deletions (indels) in ancestral sequences were inferred by maximum parsimony using PHYLIP v3.695, assuming a simple presence-absence coding scheme. As with the sequence reconstructions, each indel reconstruction was weighted by the posterior probability of the tree on which it was reconstructed.

### Structural Modeling and Docking

Structural models were constructed using MODELLER v9.10 [67], with templates selected by sequence search of the PDB database [68]. For each sequence, we constructed 100 structural models and assessed the free energy of each model using MODELLER objective function and DOPE scores [69]. We converted each model’s scores to units of deviation from the mean over the 100 models and averaged the deviation-converted MODELLER and DOPE scores to get a single overall score for each model; the model with the minimal overall score was selected as the most reliable structural model.

Hydrogen atoms were added to structural models, side-chain pKas calculated, structures optimized for favorable hydrogen bonding, and Amber force-field parameters assigned using PDB2PQR v1.7 and PROPKA v3.1 [70]. The distribution of electrostatic charge around each structure was estimated using APBS v1.4.1 [71] and projected onto the molecular surface for visualization using PyMOL.

We docked the structure of human RIG-I CARD1 (PDB ID: 4P4H) into IPS1 CARD (PDB ID: 2VGQ) using the ClusPro v2.0 [17] and Dot2 v2.0 [18] protein-protein docking protocols. For ClusPro, we used the default parameters. For Dot2, we generated and scored 54,000 orientations using a grid of 128x128x128 points with a step size of 1.0 Å and 6º rotation; other parameters had default values. We also docked structural models of human MDA5 CARD1 into IPS1 CARD using the same protocols.

## Supporting Information

S1 Fig We show the extant and ancestral protein sequences analyzed in this study, in FASTA format. GenBank identifiers are provided for extant sequences. For proteins encoding multiple caspase activation and recruitment domains (CARDs), the first (CARD1) and second (CARD2) domains are highlighted different colors. Dual-CARD proteins were expressed as single constructs for kinetics analyses. Alternative human RIG-I and MDA5 CARD1 and CARD2 domains lacking the CARD1-CARD2 linker and having boundaries determined from structural data are indicated (S5 Fig).
(TIF)

S2 Fig The first caspase activation and recruitment domains (CARDs) of human RIG-like receptors (RLRs) RIG-I and MDA5 bind the CARD domain of IPS1. We expressed and purified untagged RLR CARD constructs including the first CARD domain (CARD1), the second CARD domain (CARD2) and the N-terminal region expressing both CARD domains (CARD1+2) (Fig 1A). We measured binding of these constructs to flag-tagged IPS1 CARD using an in vitro kinetics assay (see Materials and Methods). For each kinetics experiment, we plot the shift in laser wavelength during RLR-IPS1 association (Y-axis) against the concentration of RLR CARD protein (X-axis), both at steady-state (black) and under initial conditions (blue). Bars indicate standard errors over 3 replicates. We fit one-site concentration-response curves to each set of results by nonlinear regression and estimate the ½-maximal steady-state binding concentration (Kd) and the ½-maximal initial binding rate (Km). Left-shifted curves indicate tighter binding.
(TIF)

S3 Fig Kinetics analysis of untagged RLR CARDs supports the conclusion that the first CARD domains of RIG-I and MDA5 bind tagged IPS1 CARD. We expressed and purified untagged human RLR CARD1, CARD2 and CARD1+2 constructs (Fig 1A). We measured binding of these constructs to IPS1 CARD fused to a maltose binding protein using an in vitro kinetics assay (see Materials and Methods). We plot the shift in laser wavelength during CARD-CARD association (Y-axis) against the concentration of RLR CARD (X-axis) at steady-state (black) and under initial conditions (blue). Bars indicate standard errors over 3 replicates. We fit one-site concentration-response curves by nonlinear regression and estimate the ½-maximal steady-state concentration (Kd) and ½-maximal initial binding rate (Km). Left-shifted curves indicate tighter binding. Inset, we plot the–log-transformed steady-state dissociation constant (pKd) and the–log-transformed initial binding rate (pKm) estimated over 3 replicates, with bars indicating standard errors. Human CASP9 CARD domain was used as a negative control.
(TIF)

S4 Fig Kinetics analysis of flag-tagged RLR CARDs supports the conclusion that the first CARD domains of RIG-I and MDA5 bind untagged IPS1 CARD. We purified flag-tagged RLR CARD constructs including the first CARD domain (CARD1), the second CARD domain (CARD2) and both CARD domains in tandem (CARD1+2) (Fig 1A). We measured binding of these constructs to untagged IPS1 CARD in vitro (see Materials and Methods). We plot the shift in laser wavelength during association (Y-axis) against RLR CARD concentration (X-axis) at steady-state (black) and under initial conditions (blue). Bars indicate standard errors over 3 replicates. We fit one-site concentration-response curves by nonlinear regression and estimate the ½-maximal steady-state concentration (Kd) and initial binding rate (Km). Inset, we plot the–log-transformed steady-state dissociation constant (pKd) and the–log-transformed initial binding rate (pKm). Bars indicate standard errors over 3 replicates. Human CASP9 CARD was used as a negative control.
(TIF)

S5 Fig Kinetics analysis of RLR CARD1 and CARD2 constructs matching structural domain boundaries supports the conclusion that CARD1 binds IPS1 CARD. We purified human RIG-I and MDA5 CARD1 and CARD2 structural domains, excluding CARD1-CARD2 linkers (S1 Fig). We measured binding of these constructs to IPS1 CARD in vitro (see Materials and Methods). We plot the shift in laser wavelength during association (Y-axis) against RLR CARD concentration (X-axis) at steady-state (black) and under initial conditions (blue), with bars indicating standard errors over 3 replicates. We fit one-site concentration-response curves by nonlinear regression and estimate the ½-maximal steady-state concentration (Kd) and initial binding rate (Km). Inset, we plot the–log-transformed steady-state dissociation constant (pKd) and the–log-transformed initial binding rate (pKm), with bars indicating standard errors over 3 replicates. Human CASP9 CARD was used as a negative control.
(TIF)

S6 Fig Mutations in human RIG-I CARD1 and IPS1 CARD disrupt RIG-I—IPS1 interactions in vitro. We created mutant RIG-I CARD1 (Glu66,67—Arg) and IPS1 (Arg66,67—Glu) and measured the affinities with which mutant proteins bind their wild-type partners. For each interaction, we plot the shift in laser wavelength during association (Y-axis) against RIG-I CARD1 concentration (X-axis) at steady-state (black) and under initial conditions (blue), with bars indicating standard errors over 3 replicates. We fit one-site concentration-response curves by nonlinear regression and estimate the ½-maximal steady-state concentration (Kd) and ½-maximal initial binding rate (Km), with left-shifted curves indicating tighter binding. We show binding curves for wild-type RIG-I CARD1 and CASP9 interacting with wild-type IPS1 CARD as positive and negative controls, respectively.
(TIF)

S7 Fig Prediction of the human MDA5 CARD1 –IPS1 CARD interface by protein-protein docking was ambiguous. We docked structural models of human MDA5 CARD1 into IPS1 CARD using ClusPro v2.0 (A) [17] and Dot2 v2.0 (B) [18]. Top panels show the best-scoring conformation from each analysis; bottom panels display the top 20 orientations of MDA5 CARD1 from each analysis.
(TIF)

S8 Fig Plausible evolutionary histories for RLR-IPS1 CARD domains. We plot the maximum likelihood (A) and alternative (B) phylogenies inferred from our analysis of RLR and IPS1 CARDs. Given each phylogeny, RLR gene duplication events (green circles) and duplications of individual RLR CARDs (red stars) are shown. Branch lengths are not drawn to scale.
(TIF)

S9 Fig Removal of fast-evolving non-vertebrate sequences increases support for the RLR-IPS1 CARD consensus tree. We reconstructed the RLR-IPS1 CARD phylogeny (Fig 3A) using only vertebrate and *Nematostella* CARD sequences. Support for key nodes is reported as maximum-likelihood SH-like aLRT scores (top) [61] and Bayesian posterior probabilities (bottom) [63]. RLR gene duplication events (green circles) and duplications of individual RLR CARDs (red stars) are indicated. Branch lengths are scaled to substitutions/site.
(TIF)

S10 Fig HMM-distance clustering supports the RLR-IPS1 CARD consensus phylogeny. We constructed hidden Markov models (HMMs) from separate alignments of each group of RLR and IPS1 CARDs (Fig 3) using HMMER v3.0 [24]. We then calculated the Kulback-Liebler distance between all pairs of HMMs [26] and reconstructed the neighbor joining (NJ) tree from these distances using QuickTree v1.1 [27]. We plot HMM-logo views [25] of each HMM as well as the resulting NJ tree. Branch lengths are not drawn to scale.
(TIF)

S11 Fig Gene-species tree reconciliation roots the RLR-IPS1 CARD phylogeny on *Nematostella*. We used Notung v2.6 [28, 30] to identify the optimal root for the RLR-IPS1 CARD phylogeny by reconciling the consensus RLR-IPS1 CARD tree (Fig 3A) with our current understanding of the metazoan species tree [29]. Notung uses a duplication/loss model to root gene trees in the absence of outgroup information. We plot Notung rooting scores on the consensus RLR-IPS1 CARD phylogeny. Red branches indicate optimal rootings. Pink branches indicate possible alternative roots identified by Notung (none identified). RLR gene duplication events (green circles), duplications of individual RLR CARDs (red stars) and the origin of IPS1 CARD (orange star) are indicated. Numbers in parentheses on collapsed nodes indicate the number of individual species in each group. Branch lengths are scaled to substitutions/site, inferred by maximum likelihood.
(TIF)

S12 Fig *Branchiostoma floridae* RLR CARDs interact with *B*. *floridae* IPS1 CARD in vitro. We measured the kinetics of *B*. *floridae* RIG-I CARD1+2 and MDA5 CARD1+2 domains binding to *B*. *floridae* IPS1 CARD in vitro (see Materials and Methods) (S1 Fig). We plot the shift in laser wavelength during RLR-IPS1 association (Y-axis) against RLR concentration (X-axis) at steady-state (black) and under initial conditions (blue). Bars indicate standard errors over 3 replicates. We fit one-site concentration-response curves by nonlinear regression to estimate the ½-maximal steady-state binding concentration (Kd) and the ½-maximal initial binding rate (Km). Left-shifted curves indicate tighter binding. Human CASP9 was used as a negative control.
(TIF)

S13 Fig Genomic locations of RLR and IPS1 genes in pre-vertebrate deuterostomes suggest these genes were likely clustered in the ancestral deuterostome genome. For each RLR (RIG-I, MDA5, LGP2) and IPS1 gene in the *Branchiostoma floridae* gene cluster (shown in center) (Fig 4A), we used BLAST to identify potential homologs in other pre-vertebrate deuterostome genome sequences. We identify the chromosome (for *Ciona intestinalis*) or genomic scaffold location of each BLAST hit. Colored lines indicate best-hit BLAST matches, whereas gray lines indicate alternative BLAST hits with e-values < 10^−5^.
(TIF)

S14 Fig Ancestral RLR-IPS1 CARD sequences were reconstructed with high confidence, despite ambiguity in the underlying phylogeny. We reconstructed the ancestral sequences of key nodes on the RLR-IPS1 CARD phylogeny (Fig 3A) using maximum-likelihood methods that explicitly incorporate uncertainty about the tree topology (see Materials and Methods). For each sequence, we plot the frequency with which individual residues were reconstructed with posterior probability ranging from 0.0 to 1.0, binned every 0.05 (Fig 5). Inset into each graph, we simulated protein sequence data along the maximum-likelihood phylogeny using the best-fit evolutionary model and plot the proportion of ancestral residues incorrectly inferred at each node. Dark series indicate error rates when each residue is considered unique, whereas light series indicate error rates when residues with similar biochemical properties are treated as equivalent; bars indicate standard errors. In the colored inset at top, we plot the frequency of each amino-acid residue averaged across extant sequences of each type, with bars indicating standard errors.
(TIF)

S15 Fig Simulations suggest low expected false-positive error rates for branch-sites tests of positive selection on the RLR CARD phylogeny. We used PAML v4.7 to simulate 100 replicates of codon sequence data along the maximum-likelihood RLR CARD phylogeny using three simulation models. The Empirical model estimated all simulation parameters from our empirical sequence data assuming a ‘sites’ model, in which an inferred proportion of sites evolve with nonsynonymous/synonymous rate ratio, ω<1, and the remaining sites evolve with ω = 1. The Empirical Branch-Sites model is the same as the Empirical model, except all sites are released from selective constraint (ω = 1) on the specific branch being tested. The Neutral model allows all sites to evolve neutrally (ω = 1) on all branches. All other model parameters were estimated by maximum likelihood. We analyzed each replicate data set using the same procedure used to test our empirical data for protein-coding adaptation on specific branches (see Materials and Methods). We plot the mean and standard error in false-positive error rate over the 100 data sets simulated using each model. Dotted line indicates false-positive rate of 0.05.
(TIF)

S16 Fig Ancestral RIG-like receptor (RLR) CARDs lost their capacity to bind IPS1 CARD after it diverged from RLR CARDs. We measured the binding kinetics of ancestral RLR and IPS1 CARD domains in vitro (see Materials and Methods). We plot the shift in laser wavelength during association (Y-axis) against RLR CARD concentration (X-axis) at steady-state (black) and under initial conditions (blue), with bars indicating standard errors over 3 replicates. We fit one-site concentration-response curves by nonlinear regression and estimate the ½-maximal steady-state concentration (Kd) and initial binding rate (Km). Ancestral RLR CARD binding to the ancestral IPS1 CARD progenitor before (A) and after (B) the deuterostome proliferation is shown (Fig 6). Human CASP9 CARD domain was used as a negative control.
(TIF)

S17 Fig Ancestral RIG-I and MDA5 CARD domains retained their capacity to bind the MDA5 / IPS1 CARD progenitor after they diverged from their common ancestor in deuterostomes. We measured the kinetics of ancestral RIG-I and MDA5 CARDs bound to the MDA5 CARD1 / IPS1 CARD progenitor (see Materials and Methods) (Fig 7A). We plot the shift in laser wavelength during association (Y-axis) against RLR CARD concentration (X-axis) at steady-state (black) and under initial conditions (blue), with bars indicating standard errors over 3 replicates. We fit one-site concentration-response curves by nonlinear regression and estimate the ½-maximal steady-state concentration (Kd) and ½-maximal initial binding rate (Km). Human CASP9 CARD was used as a negative control.
(TIF)

S18 Fig After the proliferation of RLR and IPS1 CARDs in deuterostomes, RIG-I CARDs diversified into IPS1-binding CARD1 and CARD2, which does not bind IPS1. We measured the kinetics of ancestral RIG-I and MDA5 CARDs bound to the ancestral IPS1 CARD after it differentiated from RLR CARDs (see Materials and Methods) (Fig 7B). We plot the shift in laser wavelength during association (Y-axis) against RLR CARD concentration (X-axis) at steady-state (black) and under initial conditions (blue), with bars indicating standard errors over 3 replicates. We fit one-site concentration-response curves by nonlinear regression and estimate the ½-maximal steady-state concentration (Kd) and ½-maximal initial binding rate (Km). Human CASP9 CARD was used as a negative control.
(TIF)

S1 Table Gene conversion is rare among extant RIG-like receptor (RLR) caspase activation and recruitment domains (CARDs). We used GENECONV v1.81a to identify regions of potential gene conversion among RLR CARDs [32]. We report results with significant support for gene conversion between sequence pairs, after correcting for multiple tests (*p*<0.05). **Sim P**, simulated *p* values based on 10,000 permutations; **BC KA P**, Bonferroni-corrected Karlin–Altschul *p* values; **Beg**, first nucleotide of the potential converted region; **End**, last nucleotide of the potential converted region; **Poly**, number of polymorphic sites in the region; **Len**, length of the converted region; **Diff**; number of nucleotide mismatches in the potential converted region; **Total Diff**, total number of nucleotide mismatches between the two sequences.
(DOCX)
