# Supplementary figures and images for: Evolution of a Novel Antiviral Immune-Signaling Interaction by Partial-Gene Duplication
Source: PLoS One. 2015 Sep 10;10(9):e0137276. doi: 10.1371/journal.pone.0137276 (PMC4565553; doi:10.1371/journal.pone.0137276)

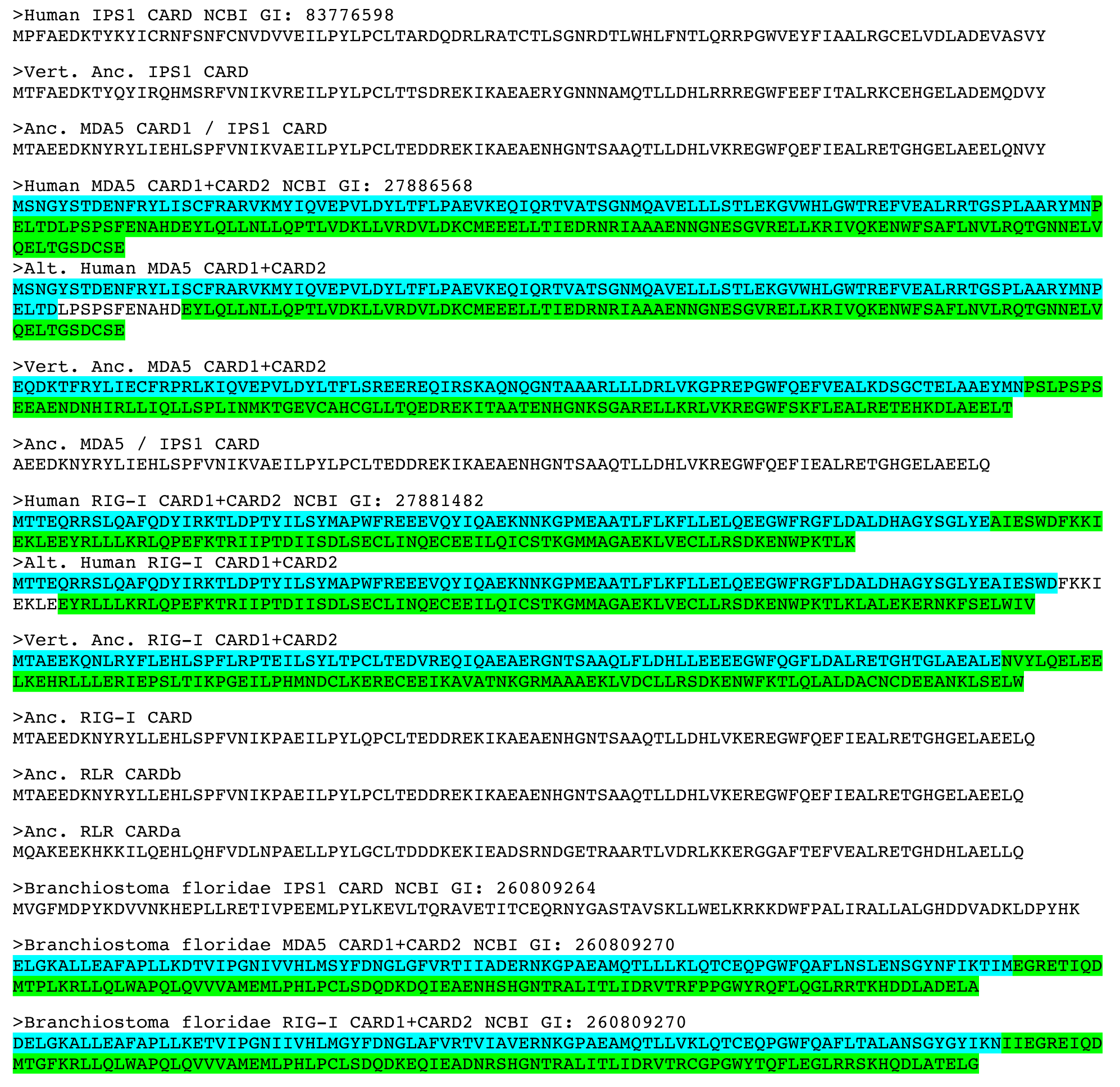

Supplement: S1 Fig — GenBank identifiers are provided for extant sequences. For proteins encoding multiple caspase activation and recruitment domains (CARDs), the first (CARD1) and second (CARD2) domains are highlighted different colors. Dual-CARD proteins were expressed as single constructs for kinetics analyses. Alternative human RIG-I and MDA5 CARD1 and CARD2 domains lacking the CARD1-CARD2 linker and having boundaries determined from structural data are indicated (S5 Fig). (TIF) [file pone.0137276.s001.tif]

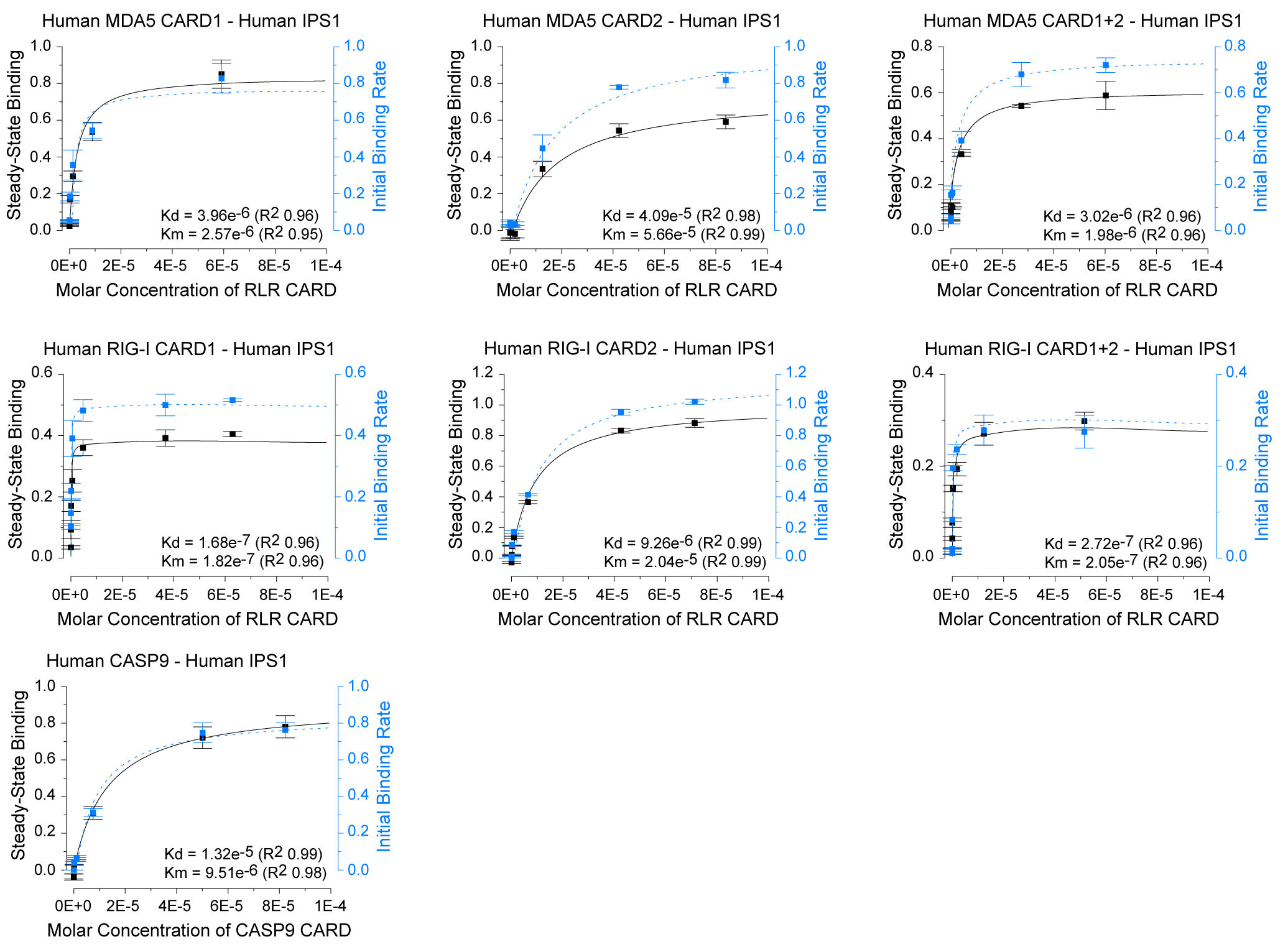

Supplement: S2 Fig — We expressed and purified untagged RLR CARD constructs including the first CARD domain (CARD1), the second CARD domain (CARD2) and the N-terminal region expressing both CARD domains (CARD1+2) (Fig 1A). We measured binding of these constructs to flag-tagged IPS1 CARD using an in vitro kinetics assay (see Materials and Methods). For each kinetics experiment, we plot the shift in laser wavelength during RLR-IPS1 association (Y-axis) against the concentration of RLR CARD protein (X-axis), both at steady-state (black) and under initial conditions (blue). Bars indicate standard errors over 3 replicates. We fit one-site concentration-response curves to each set of results by nonlinear regression and estimate the ½-maximal steady-state binding concentration (Kd) and the ½-maximal initial binding rate (Km). Left-shifted curves indicate tighter binding. (TIF) [file pone.0137276.s002.tif]

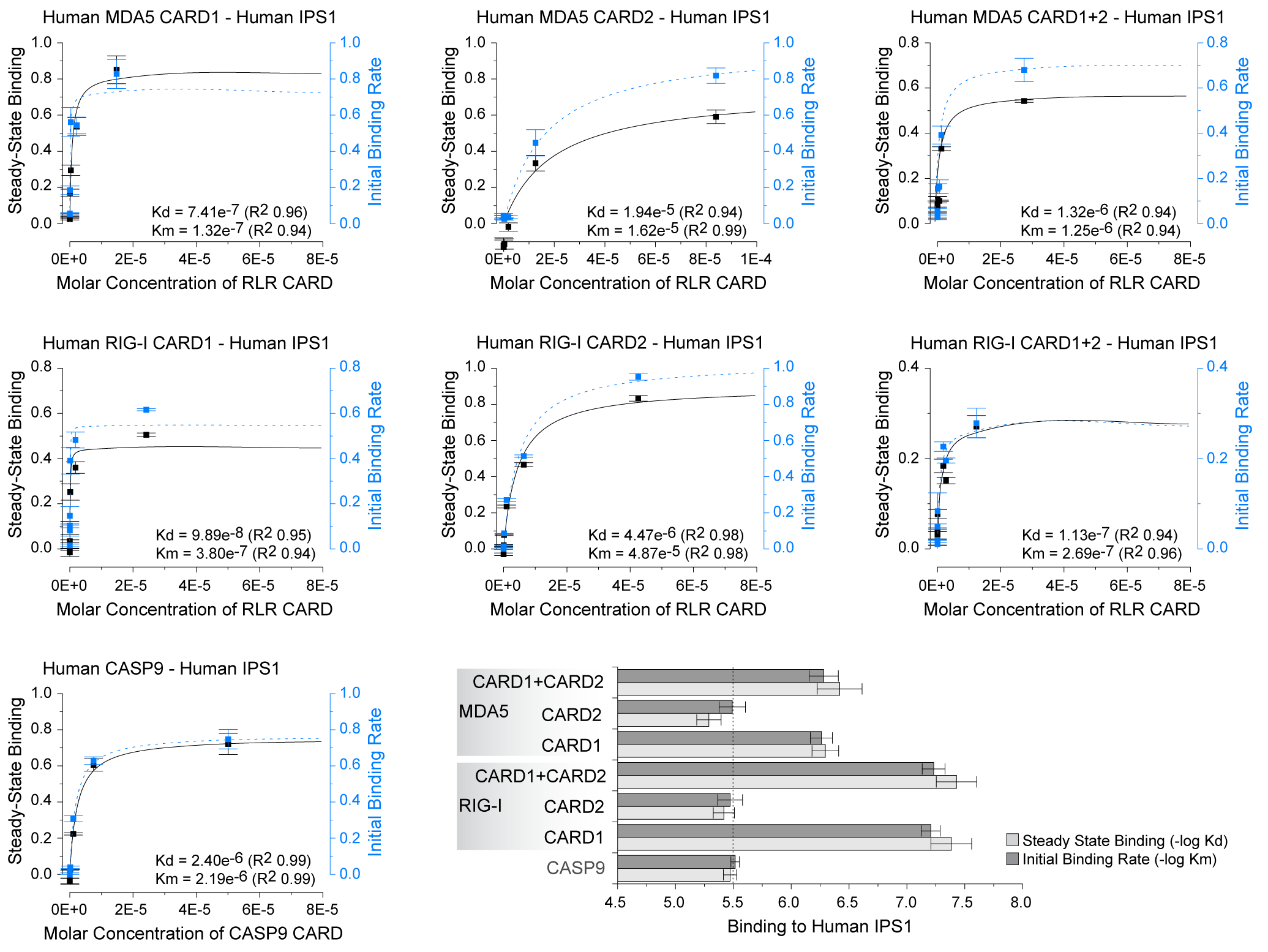

Supplement: S3 Fig — We expressed and purified untagged human RLR CARD1, CARD2 and CARD1+2 constructs (Fig 1A). We measured binding of these constructs to IPS1 CARD fused to a maltose binding protein using an in vitro kinetics assay (see Materials and Methods). We plot the shift in laser wavelength during CARD-CARD association (Y-axis) against the concentration of RLR CARD (X-axis) at steady-state (black) and under initial conditions (blue). Bars indicate standard errors over 3 replicates. We fit one-site concentration-response curves by nonlinear regression and estimate the ½-maximal steady-state concentration (Kd) and ½-maximal initial binding rate (Km). Left-shifted curves indicate tighter binding. Inset, we plot the–log-transformed steady-state dissociation constant (pKd) and the–log-transformed initial binding rate (pKm) estimated over 3 replicates, with bars indicating standard errors. Human CASP9 CARD domain was used as a negative control. (TIF) [file pone.0137276.s003.tif]

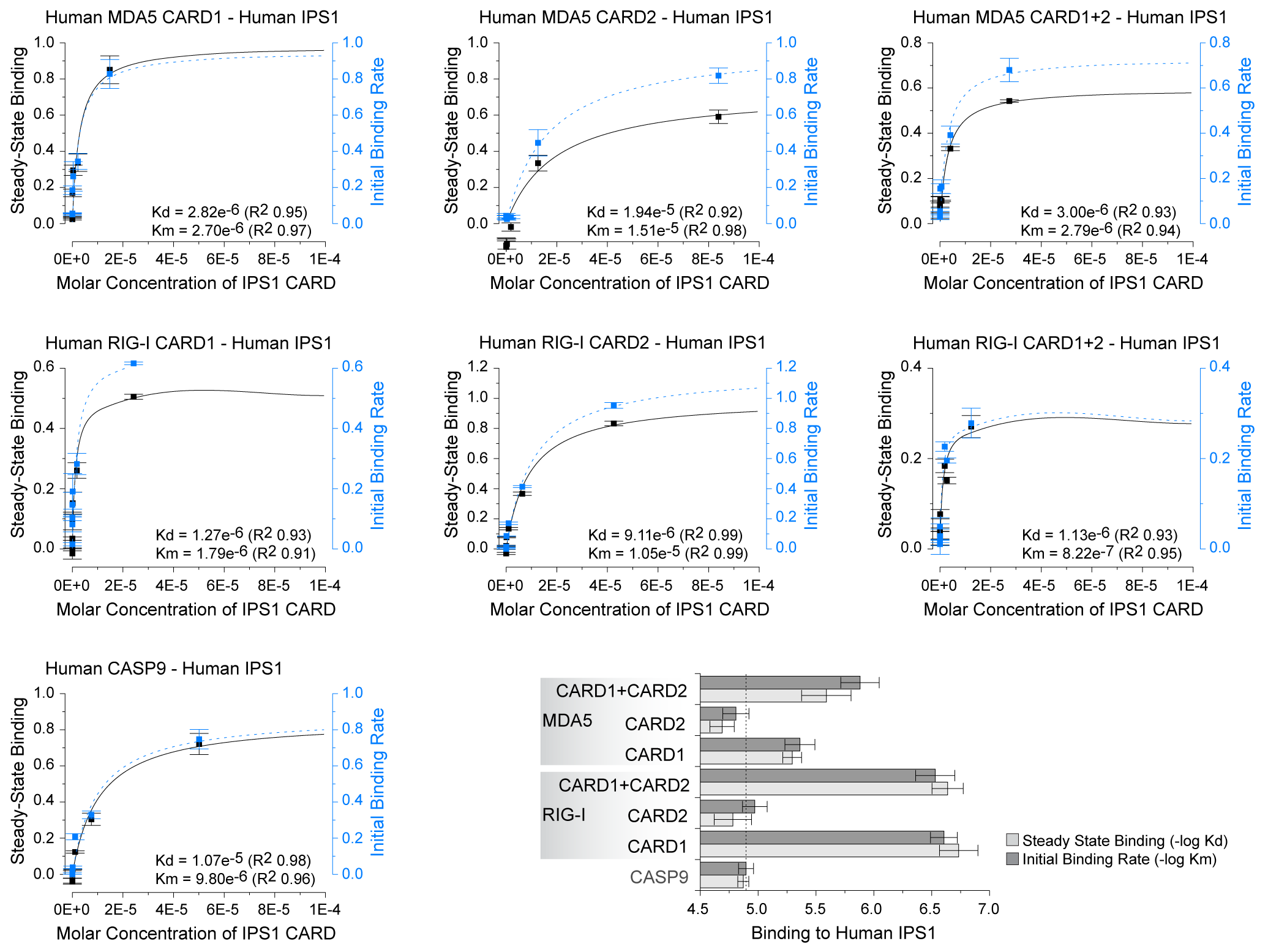

Supplement: S4 Fig — We purified flag-tagged RLR CARD constructs including the first CARD domain (CARD1), the second CARD domain (CARD2) and both CARD domains in tandem (CARD1+2) (Fig 1A). We measured binding of these constructs to untagged IPS1 CARD in vitro (see Materials and Methods). We plot the shift in laser wavelength during association (Y-axis) against RLR CARD concentration (X-axis) at steady-state (black) and under initial conditions (blue). Bars indicate standard errors over 3 replicates. We fit one-site concentration-response curves by nonlinear regression and estimate the ½-maximal steady-state concentration (Kd) and initial binding rate (Km). Inset, we plot the–log-transformed steady-state dissociation constant (pKd) and the–log-transformed initial binding rate (pKm). Bars indicate standard errors over 3 replicates. Human CASP9 CARD was used as a negative control. (TIF) [file pone.0137276.s004.tif]

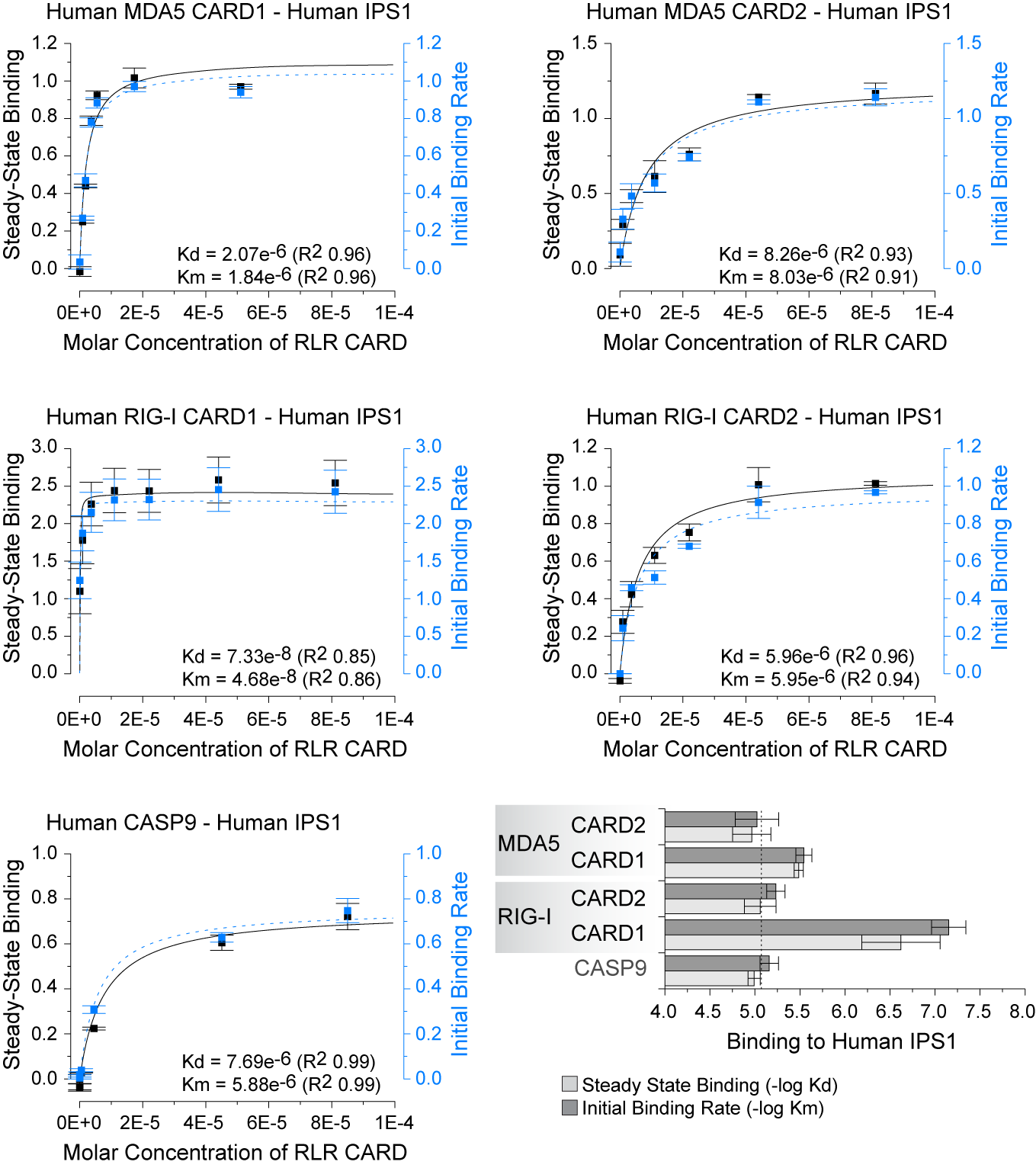

Supplement: S5 Fig — We purified human RIG-I and MDA5 CARD1 and CARD2 structural domains, excluding CARD1-CARD2 linkers (S1 Fig). We measured binding of these constructs to IPS1 CARD in vitro (see Materials and Methods). We plot the shift in laser wavelength during association (Y-axis) against RLR CARD concentration (X-axis) at steady-state (black) and under initial conditions (blue), with bars indicating standard errors over 3 replicates. We fit one-site concentration-response curves by nonlinear regression and estimate the ½-maximal steady-state concentration (Kd) and initial binding rate (Km). Inset, we plot the–log-transformed steady-state dissociation constant (pKd) and the–log-transformed initial binding rate (pKm), with bars indicating standard errors over 3 replicates. Human CASP9 CARD was used as a negative control. (TIF) [file pone.0137276.s005.tif]

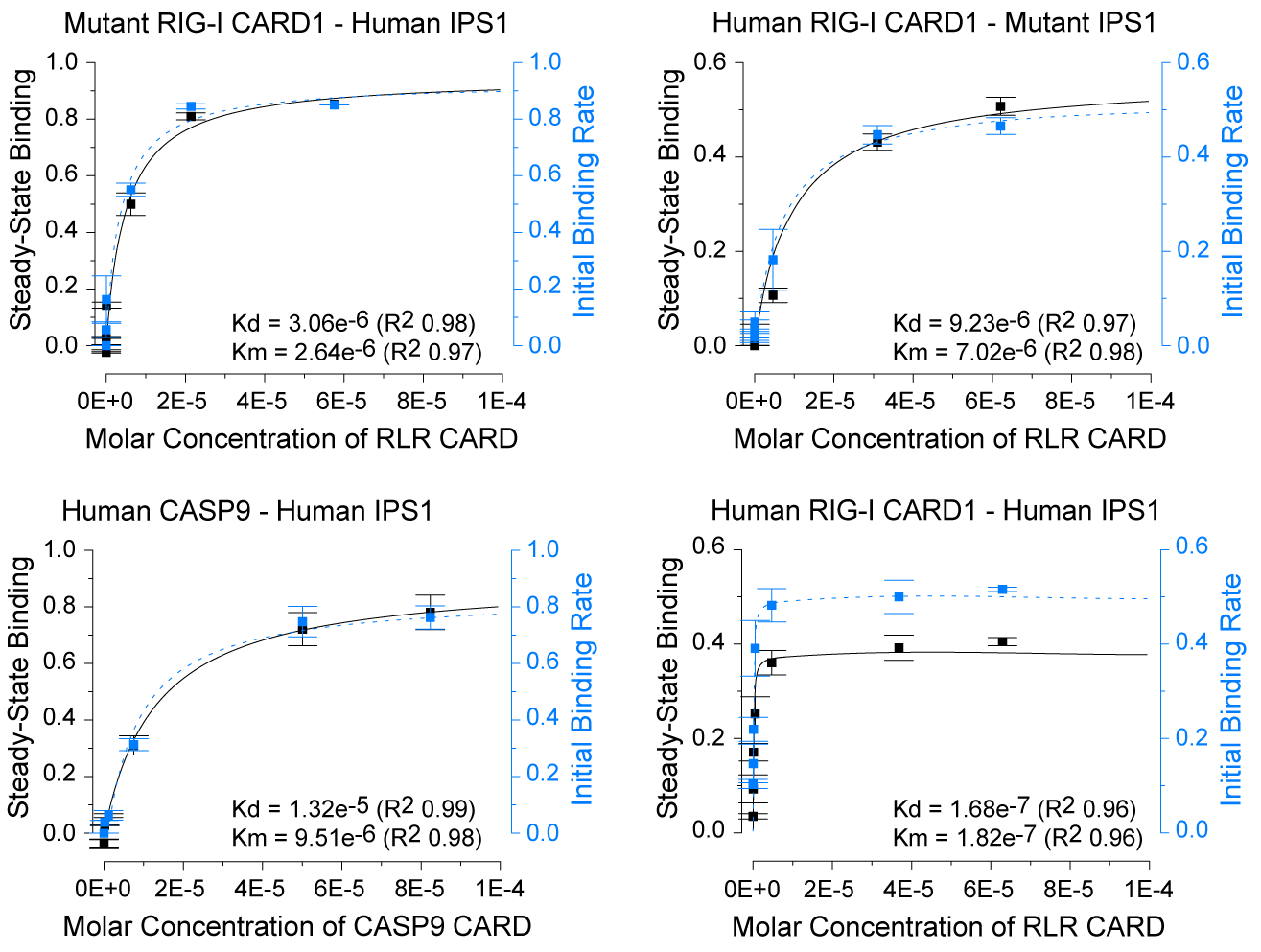

Supplement: S6 Fig — We created mutant RIG-I CARD1 (Glu66,67—Arg) and IPS1 (Arg66,67—Glu) and measured the affinities with which mutant proteins bind their wild-type partners. For each interaction, we plot the shift in laser wavelength during association (Y-axis) against RIG-I CARD1 concentration (X-axis) at steady-state (black) and under initial conditions (blue), with bars indicating standard errors over 3 replicates. We fit one-site concentration-response curves by nonlinear regression and estimate the ½-maximal steady-state concentration (Kd) and ½-maximal initial binding rate (Km), with left-shifted curves indicating tighter binding. We show binding curves for wild-type RIG-I CARD1 and CASP9 interacting with wild-type IPS1 CARD as positive and negative controls, respectively. (TIF) [file pone.0137276.s006.tif]

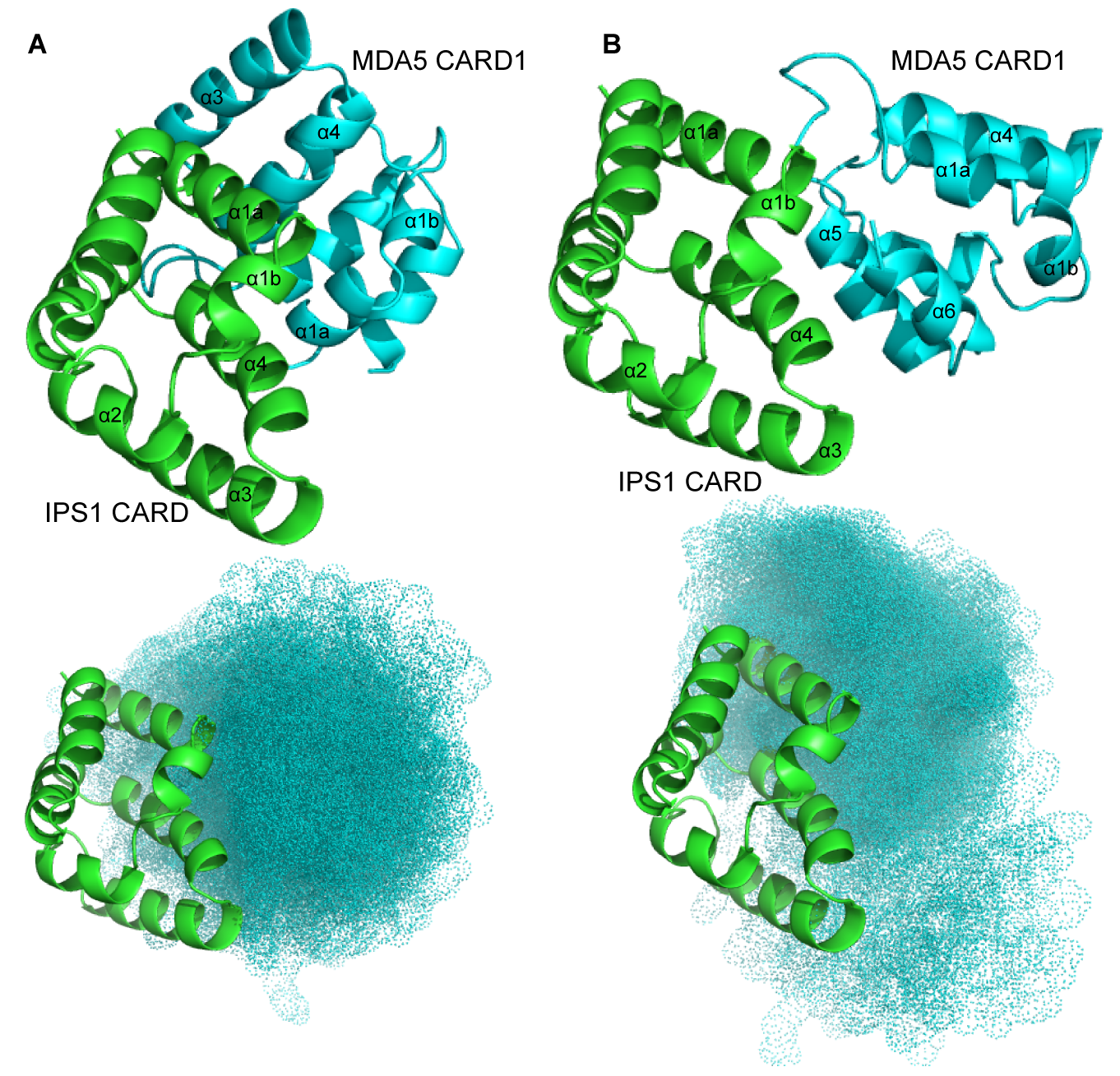

Supplement: S7 Fig — We docked structural models of human MDA5 CARD1 into IPS1 CARD using ClusPro v2.0 (A) [17] and Dot2 v2.0 (B) [18]. Top panels show the best-scoring conformation from each analysis; bottom panels display the top 20 orientations of MDA5 CARD1 from each analysis. (TIF) [file pone.0137276.s007.tif]

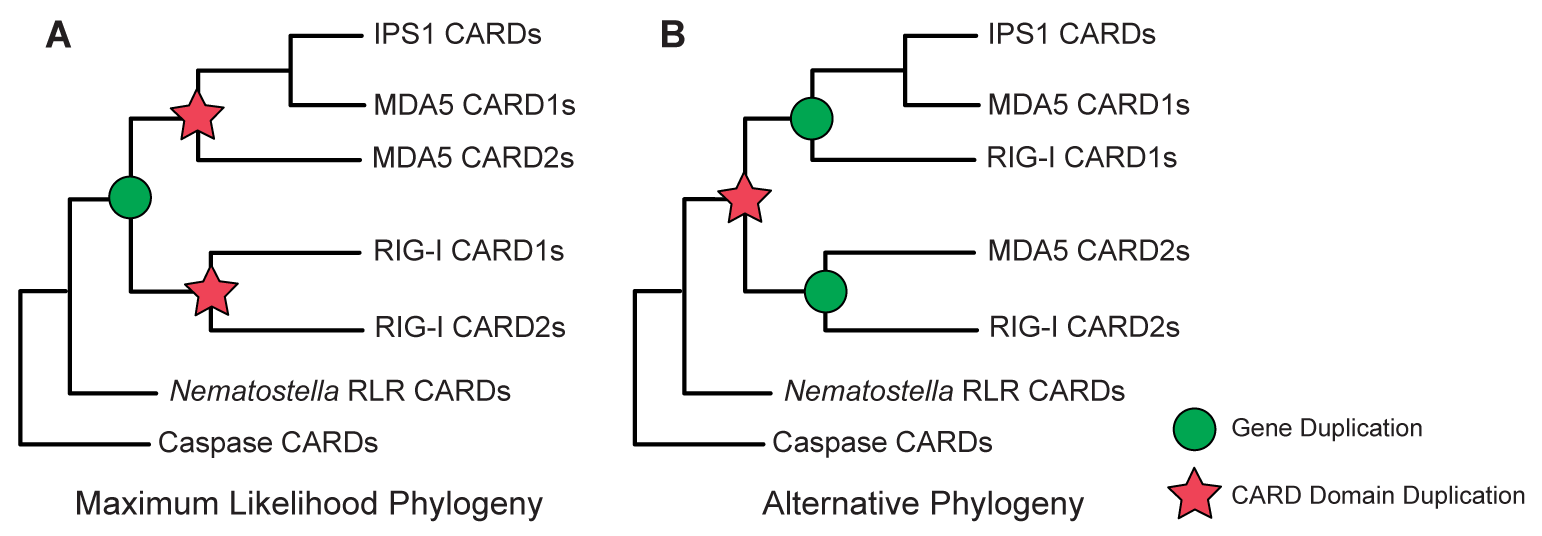

Supplement: S8 Fig — We plot the maximum likelihood (A) and alternative (B) phylogenies inferred from our analysis of RLR and IPS1 CARDs. Given each phylogeny, RLR gene duplication events (green circles) and duplications of individual RLR CARDs (red stars) are shown. Branch lengths are not drawn to scale. (TIF) [file pone.0137276.s008.tif]

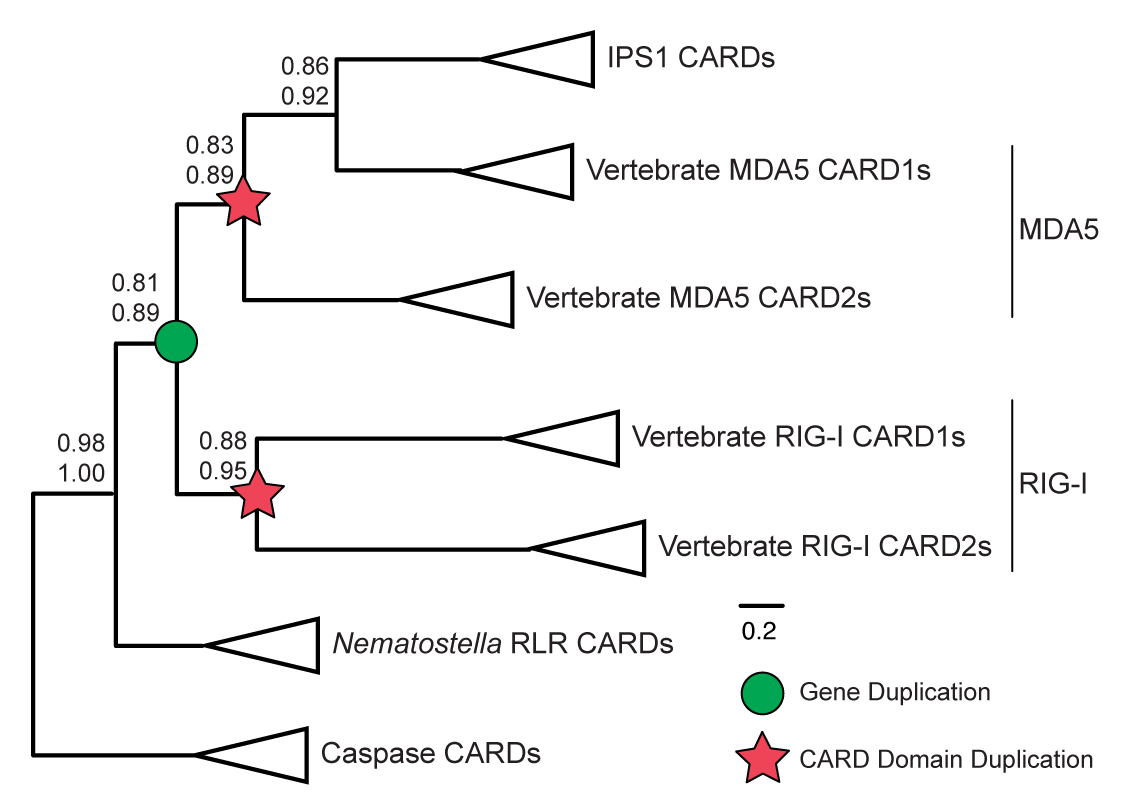

Supplement: S9 Fig — We reconstructed the RLR-IPS1 CARD phylogeny (Fig 3A) using only vertebrate and Nematostella CARD sequences. Support for key nodes is reported as maximum-likelihood SH-like aLRT scores (top) [61] and Bayesian posterior probabilities (bottom) [63]. RLR gene duplication events (green circles) and duplications of individual RLR CARDs (red stars) are indicated. Branch lengths are scaled to substitutions/site. (TIF) [file pone.0137276.s009.tif]

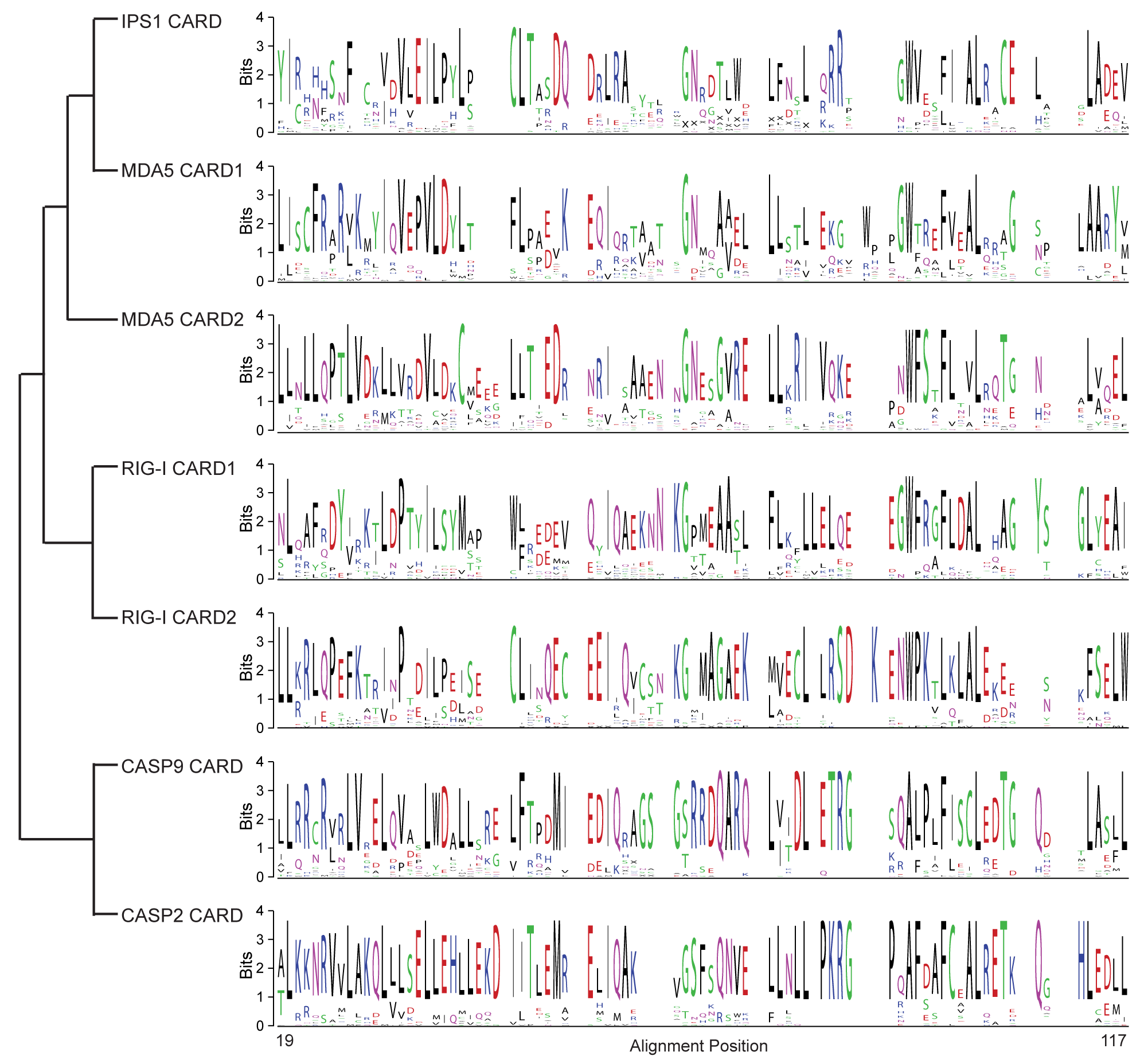

Supplement: S10 Fig — We constructed hidden Markov models (HMMs) from separate alignments of each group of RLR and IPS1 CARDs (Fig 3) using HMMER v3.0 [24]. We then calculated the Kulback-Liebler distance between all pairs of HMMs [26] and reconstructed the neighbor joining (NJ) tree from these distances using QuickTree v1.1 [27]. We plot HMM-logo views [25] of each HMM as well as the resulting NJ tree. Branch lengths are not drawn to scale. (TIF) [file pone.0137276.s010.tif]

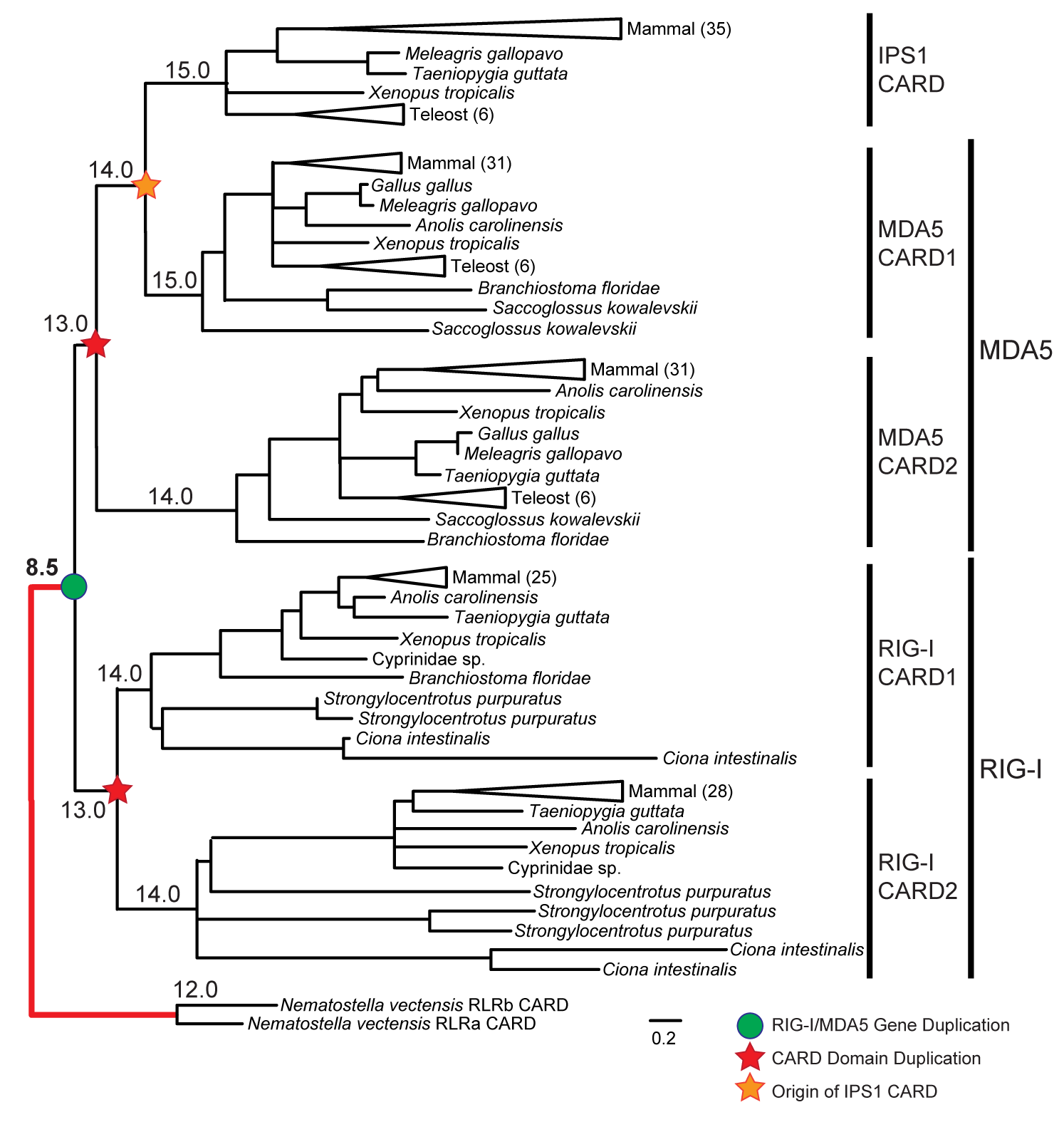

Supplement: S11 Fig — We used Notung v2.6 [28, 30] to identify the optimal root for the RLR-IPS1 CARD phylogeny by reconciling the consensus RLR-IPS1 CARD tree (Fig 3A) with our current understanding of the metazoan species tree [29]. Notung uses a duplication/loss model to root gene trees in the absence of outgroup information. We plot Notung rooting scores on the consensus RLR-IPS1 CARD phylogeny. Red branches indicate optimal rootings. Pink branches indicate possible alternative roots identified by Notung (none identified). RLR gene duplication events (green circles), duplications of individual RLR CARDs (red stars) and the origin of IPS1 CARD (orange star) are indicated. Numbers in parentheses on collapsed nodes indicate the number of individual species in each group. Branch lengths are scaled to substitutions/site, inferred by maximum likelihood. (TIF) [file pone.0137276.s011.tif]

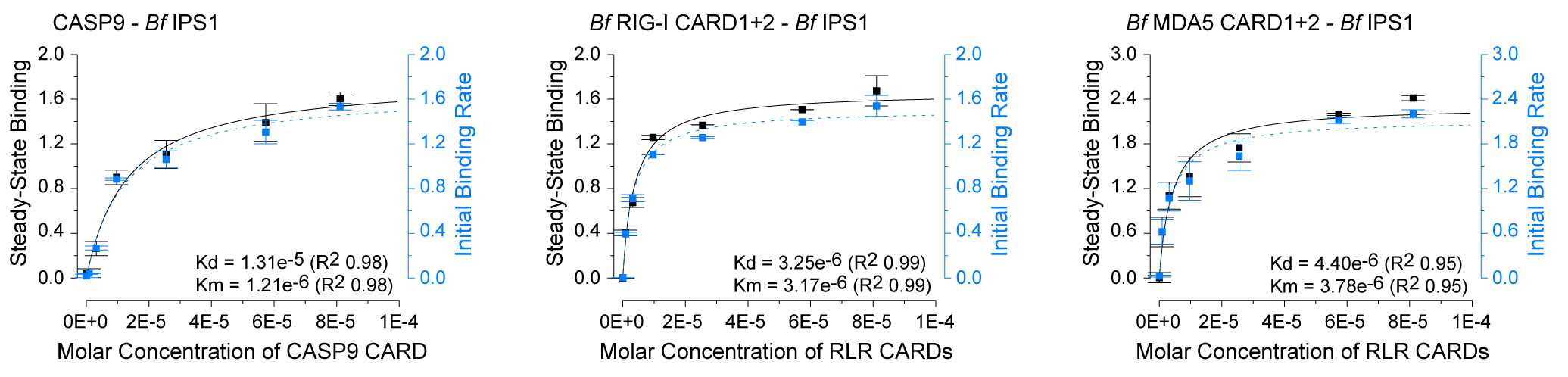

Supplement: S12 Fig — We measured the kinetics of B. floridae RIG-I CARD1+2 and MDA5 CARD1+2 domains binding to B. floridae IPS1 CARD in vitro (see Materials and Methods) (S1 Fig). We plot the shift in laser wavelength during RLR-IPS1 association (Y-axis) against RLR concentration (X-axis) at steady-state (black) and under initial conditions (blue). Bars indicate standard errors over 3 replicates. We fit one-site concentration-response curves by nonlinear regression to estimate the ½-maximal steady-state binding concentration (Kd) and the ½-maximal initial binding rate (Km). Left-shifted curves indicate tighter binding. Human CASP9 was used as a negative control. (TIF) [file pone.0137276.s012.tif]

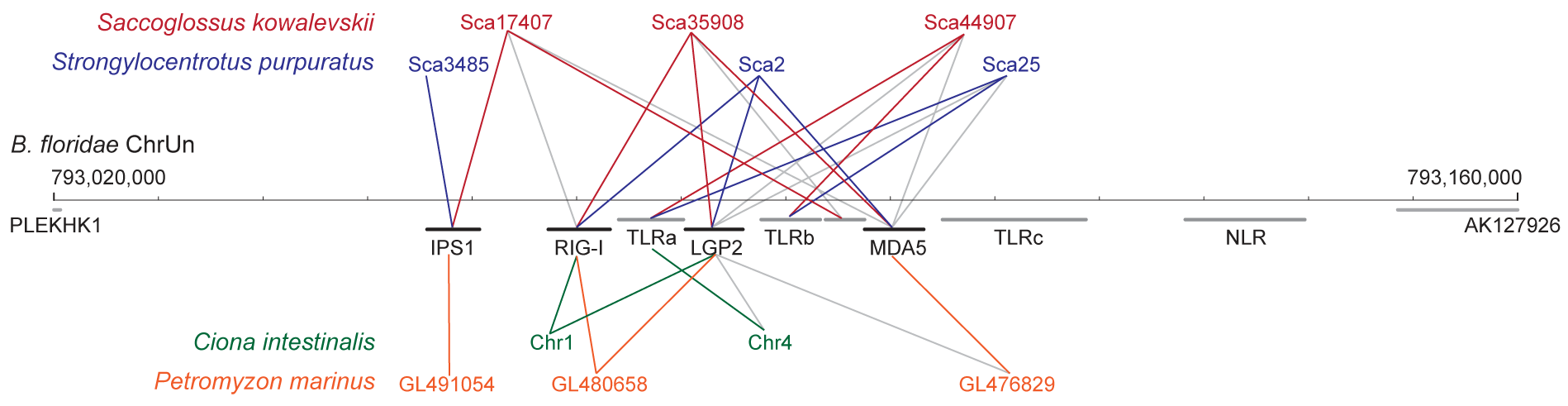

Supplement: S13 Fig — For each RLR (RIG-I, MDA5, LGP2) and IPS1 gene in the Branchiostoma floridae gene cluster (shown in center) (Fig 4A), we used BLAST to identify potential homologs in other pre-vertebrate deuterostome genome sequences. We identify the chromosome (for Ciona intestinalis) or genomic scaffold location of each BLAST hit. Colored lines indicate best-hit BLAST matches, whereas gray lines indicate alternative BLAST hits with e-values < 10−5. (TIF) [file pone.0137276.s013.tif]

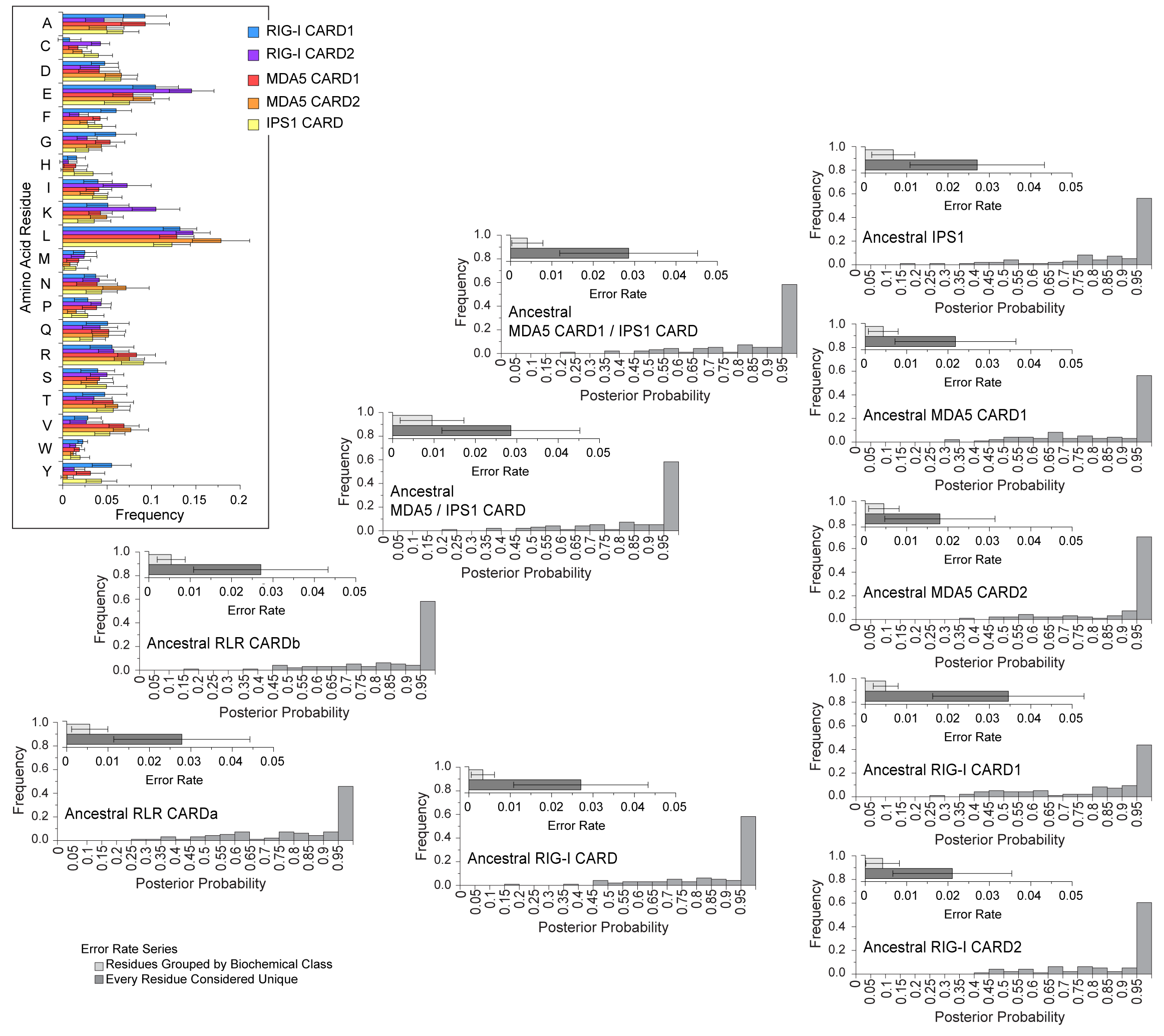

Supplement: S14 Fig — We reconstructed the ancestral sequences of key nodes on the RLR-IPS1 CARD phylogeny (Fig 3A) using maximum-likelihood methods that explicitly incorporate uncertainty about the tree topology (see Materials and Methods). For each sequence, we plot the frequency with which individual residues were reconstructed with posterior probability ranging from 0.0 to 1.0, binned every 0.05 (Fig 5). Inset into each graph, we simulated protein sequence data along the maximum-likelihood phylogeny using the best-fit evolutionary model and plot the proportion of ancestral residues incorrectly inferred at each node. Dark series indicate error rates when each residue is considered unique, whereas light series indicate error rates when residues with similar biochemical properties are treated as equivalent; bars indicate standard errors. In the colored inset at top, we plot the frequency of each amino-acid residue averaged across extant sequences of each type, with bars indicating standard errors. (TIF) [file pone.0137276.s014.tif]

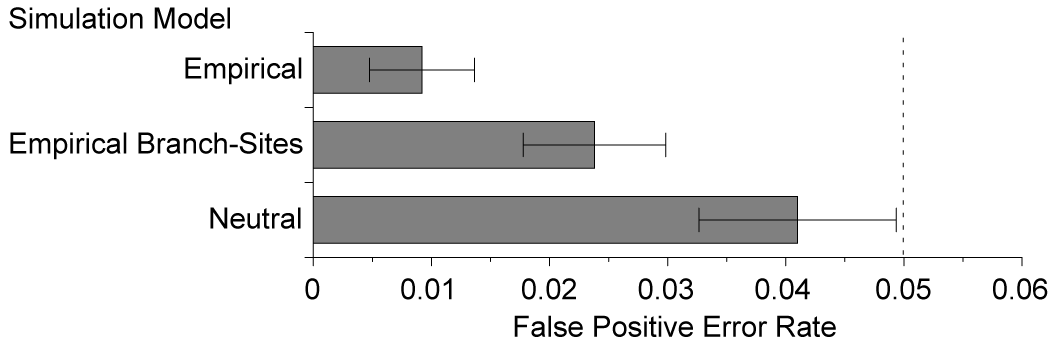

Supplement: S15 Fig — We used PAML v4.7 to simulate 100 replicates of codon sequence data along the maximum-likelihood RLR CARD phylogeny using three simulation models. The Empirical model estimated all simulation parameters from our empirical sequence data assuming a ‘sites’ model, in which an inferred proportion of sites evolve with nonsynonymous/synonymous rate ratio, ω<1, and the remaining sites evolve with ω = 1. The Empirical Branch-Sites model is the same as the Empirical model, except all sites are released from selective constraint (ω = 1) on the specific branch being tested. The Neutral model allows all sites to evolve neutrally (ω = 1) on all branches. All other model parameters were estimated by maximum likelihood. We analyzed each replicate data set using the same procedure used to test our empirical data for protein-coding adaptation on specific branches (see Materials and Methods). We plot the mean and standard error in false-positive error rate over the 100 data sets simulated using each model. Dotted line indicates false-positive rate of 0.05. (TIF) [file pone.0137276.s015.tif]

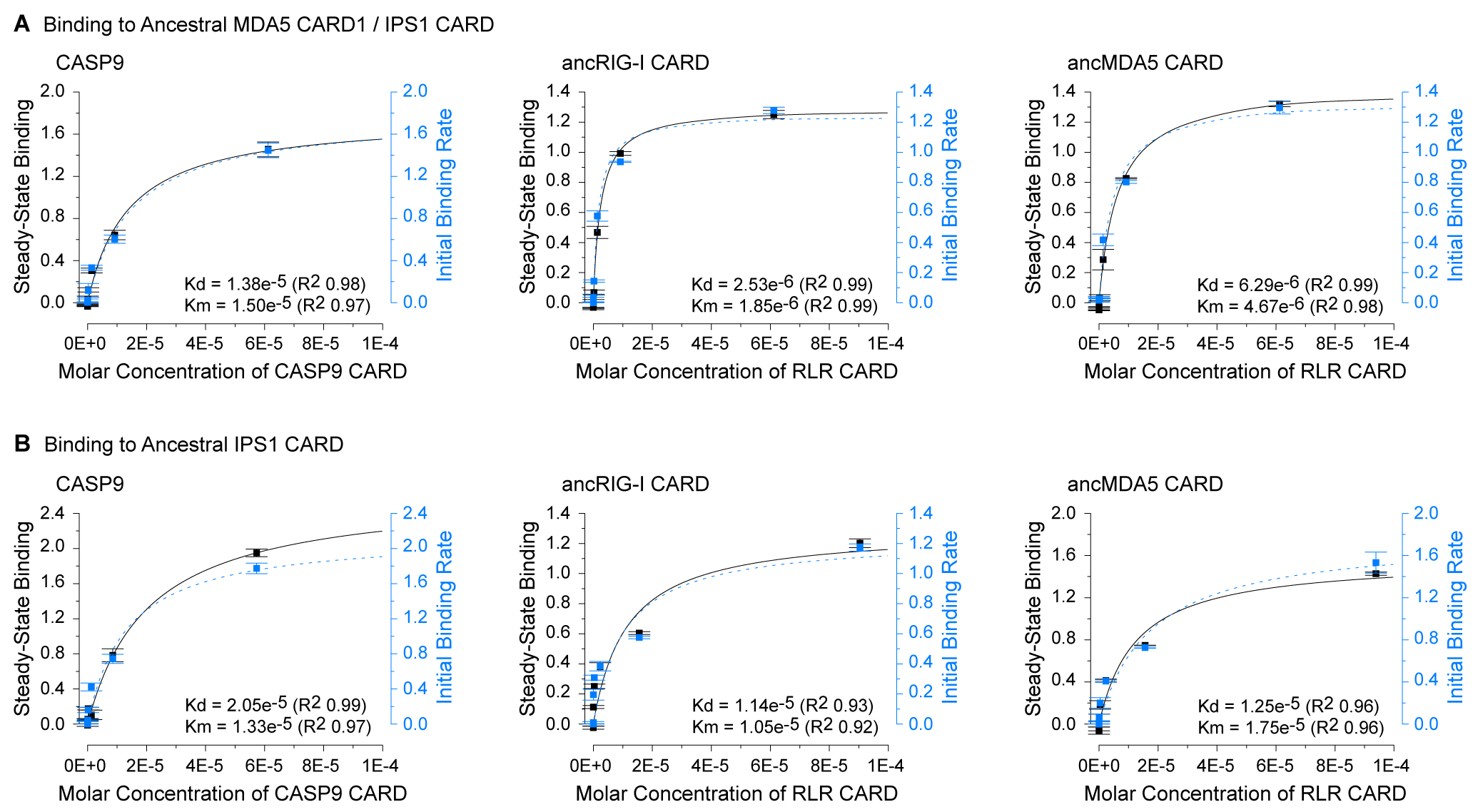

Supplement: S16 Fig — We measured the binding kinetics of ancestral RLR and IPS1 CARD domains in vitro (see Materials and Methods). We plot the shift in laser wavelength during association (Y-axis) against RLR CARD concentration (X-axis) at steady-state (black) and under initial conditions (blue), with bars indicating standard errors over 3 replicates. We fit one-site concentration-response curves by nonlinear regression and estimate the ½-maximal steady-state concentration (Kd) and initial binding rate (Km). Ancestral RLR CARD binding to the ancestral IPS1 CARD progenitor before (A) and after (B) the deuterostome proliferation is shown (Fig 6). Human CASP9 CARD domain was used as a negative control. (TIF) [file pone.0137276.s016.tif]

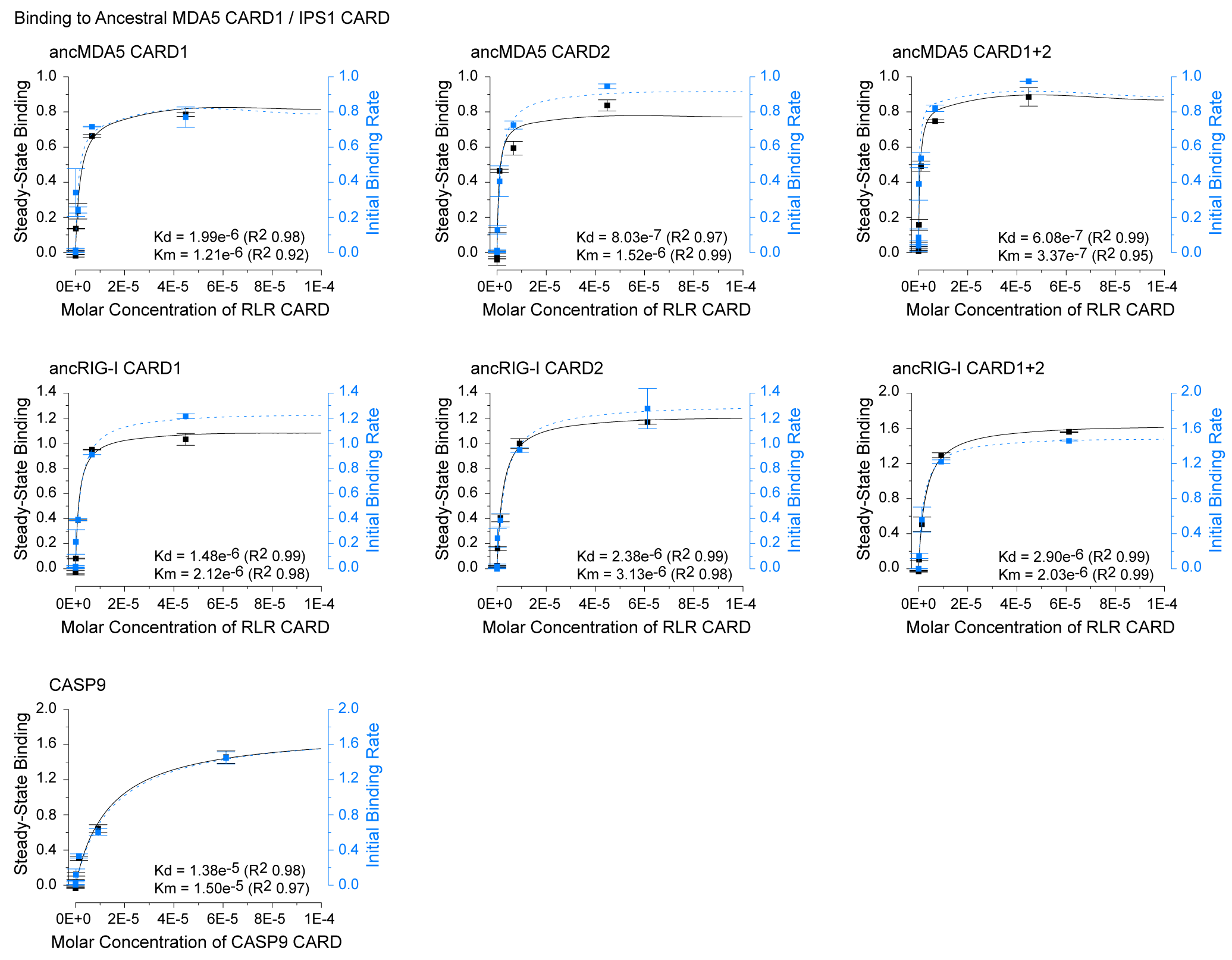

Supplement: S17 Fig — We measured the kinetics of ancestral RIG-I and MDA5 CARDs bound to the MDA5 CARD1 / IPS1 CARD progenitor (see Materials and Methods) (Fig 7A). We plot the shift in laser wavelength during association (Y-axis) against RLR CARD concentration (X-axis) at steady-state (black) and under initial conditions (blue), with bars indicating standard errors over 3 replicates. We fit one-site concentration-response curves by nonlinear regression and estimate the ½-maximal steady-state concentration (Kd) and ½-maximal initial binding rate (Km). Human CASP9 CARD was used as a negative control. (TIF) [file pone.0137276.s017.tif]

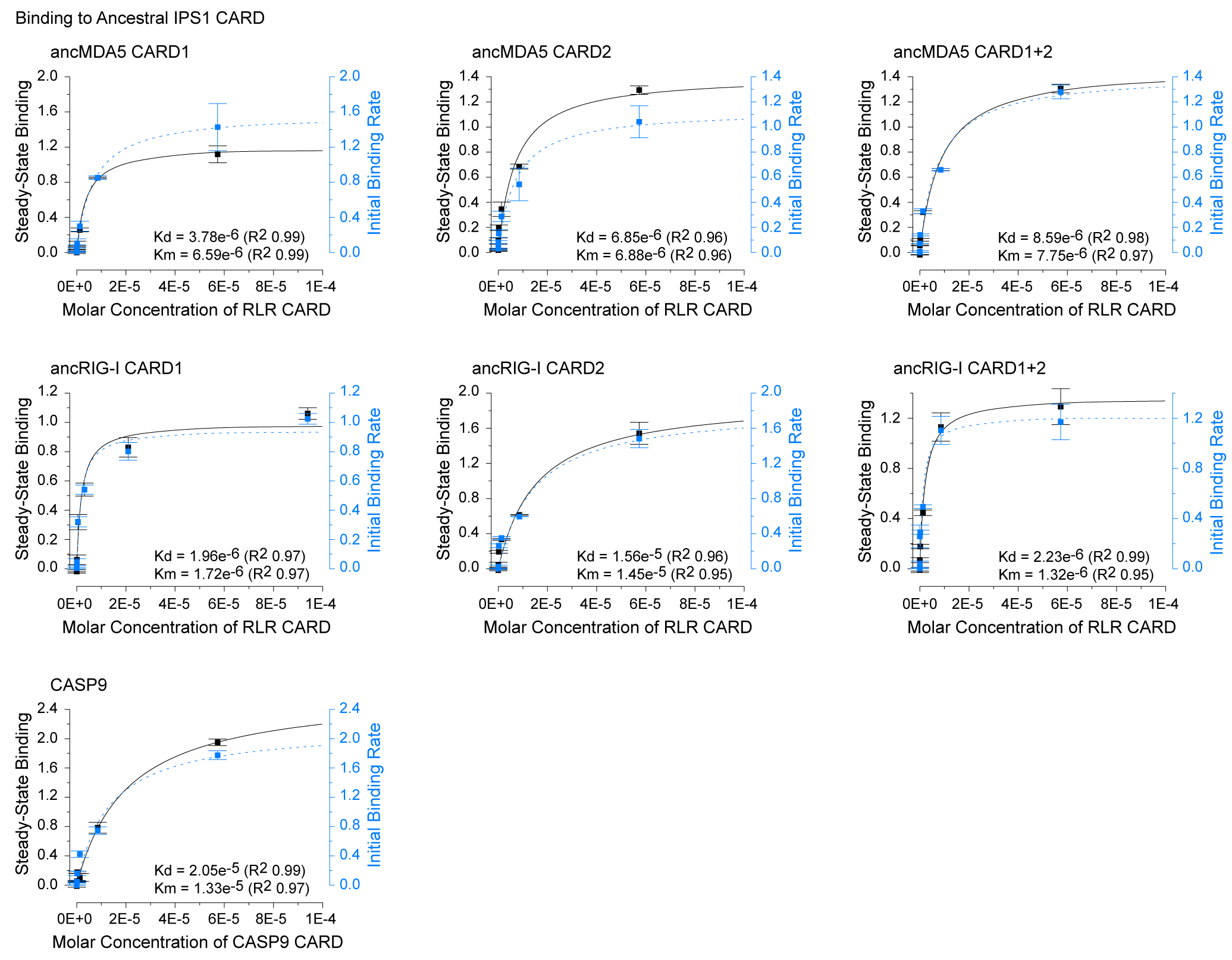

Supplement: S18 Fig — We measured the kinetics of ancestral RIG-I and MDA5 CARDs bound to the ancestral IPS1 CARD after it differentiated from RLR CARDs (see Materials and Methods) (Fig 7B). We plot the shift in laser wavelength during association (Y-axis) against RLR CARD concentration (X-axis) at steady-state (black) and under initial conditions (blue), with bars indicating standard errors over 3 replicates. We fit one-site concentration-response curves by nonlinear regression and estimate the ½-maximal steady-state concentration (Kd) and ½-maximal initial binding rate (Km). Human CASP9 CARD was used as a negative control. (TIF) [file pone.0137276.s018.tif]
