# Supplementary material for: Evolution of a Novel Antiviral Immune-Signaling Interaction by Partial-Gene Duplication
Source: PLoS One. 2015 Sep 10;10(9):e0137276. doi: 10.1371/journal.pone.0137276 (PMC4565553; doi:10.1371/journal.pone.0137276)
Supplement: S1 Table — We used GENECONV v1.81a to identify regions of potential gene conversion among RLR CARDs [32]. We report results with significant support for gene conversion between sequence pairs, after correcting for multiple tests (p<0.05). Sim P, simulated p values based on 10,000 permutations; BC KA P, Bonferroni-corrected Karlin–Altschul p values; Beg, first nucleotide of the potential converted region; End, last nucleotide of the potential converted region; Poly, number of polymorphic sites in the region; Len, length of the converted region; Diff; number of nucleotide mismatches in the potential converted region; Total Diff, total number of nucleotide mismatches between the two sequences. (DOCX) [file pone.0137276.s019.docx]

| **Sequence Pair** | **Sim P** | **BC KA P** | **Beg** | **End** | **Len** | **Poly** | **Diff** | **Total Diff** |
| --- | --- | --- | --- | --- | --- | --- | --- | --- |
| *Pteropus vampyrus* MDA5 CARD2; *Ailuropoda melanoleucaI* MDA5 CARD2 | 0.0197 | 0.4036 | 200 | 276 | 77 | 54 | 0 | 33 |
| *Myotis lucifugus* MDA5 CARD2; *Pongo abelii* MDA5 CARD2 | 0.0292 | 0.5414 | 238 | 278 | 41 | 25 | 0 | 63 |
